# Carbon Dioxide Capture and Conversion Using Metal–Organic Framework (MOF) Materials: A Comprehensive Review

**DOI:** 10.3390/nano14161340

**Published:** 2024-08-12

**Authors:** Fanyi Kong, Wenqian Chen

**Affiliations:** Key Laboratory of Organic Compound Pollution Control Engineering (MOE), School of Environmental and Chemical Engineering, Shanghai University, Shanghai 200444, China; 2622747409@shu.edu.cn

**Keywords:** MOF-based materials, CO_2_ capture, CO_2_ conversion

## Abstract

The escalating threat of anthropogenic climate change has spurred an urgent quest for innovative CO_2_ capture and utilization (CCU) technologies. Metal–organic frameworks (MOFs) have emerged as prominent candidates in CO_2_ capture and conversion due to their large specific surface area, well-defined porous structure, and tunable chemical properties. This review unveils the latest advancements in MOF-based materials specifically designed for superior CO_2_ adsorption, precise separation, advanced photocatalytic and electrocatalytic CO_2_ reduction, progressive CO_2_ hydrogenation, and dual functionalities. We explore the strategies that enhance MOF efficiency and examine the challenges of and opportunities afforded by transitioning from laboratory research to industrial application. Looking ahead, this review offers a visionary perspective on harnessing MOFs for the sustainable capture and conversion of CO_2_.

## 1. Introduction

Climate change is one of the most critical global challenges and necessitates immediate attention. The continuous increase in greenhouse gas emissions, particularly CO_2_, has triggered a series of environmental problems, including extreme weather events, forest disturbances, and water erosion [1,2,3,4]. These issues significantly impact human society, as evidenced by the heightened incidences of infectious diseases and intensified rainfall in recent years [5,6,7].

Of particular concern is the rapid escalation in global surface temperatures, surpassing the rates observed over any other 50-year period in the past two millennia. This alarming trend is expected to endure, with a potential to exert further detrimental influence on human civilization. The deleterious impacts of climate change transcend mere ecological harm, extending to food security, biodiversity, economy, and cultural heritage [8,9,10]. Central to these widespread concerns is the continuous rise of the greenhouse gas CO_2_.

**Figure 1:**
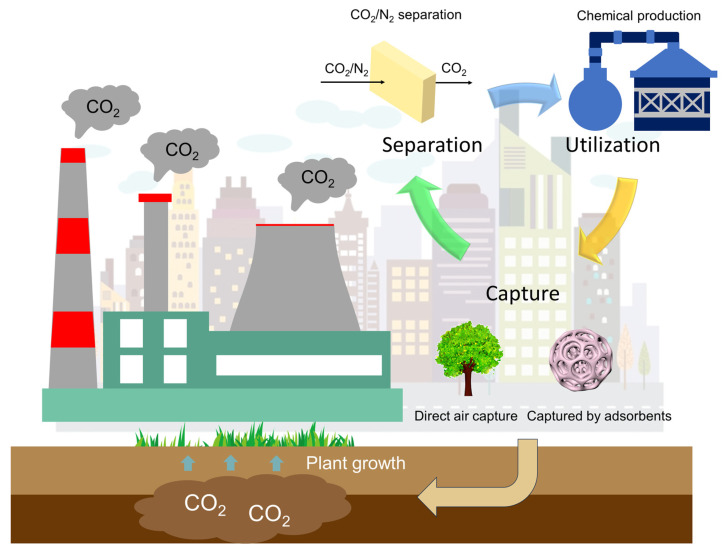
Schematic illustration of the CCU technology.

In the endeavor to mitigate the environmental impact of CO_2_, enormous efforts have been dedicated toward accelerating the transition to sustainable energy sources and advancing traditional technologies. Among the approaches developed is carbon capture and utilization (CCU) technology, entailing the capture of CO_2_ from the atmosphere or from fuel gases and conversion into value-added industrial products (Figure 1). CCU has emerged as a promising technology in reducing the concentration of CO_2_ in the atmosphere [11]. The key to this technology hinges on the development of high-performance and cost-effective materials serving as sorbents or catalysts for CO_2_ capture and conversion. Various materials have been explored for CO_2_ capture and conversion, with solid materials being the most prominent due to their cost-effectiveness and flexible structures [12]. Notable examples include the following:

Activated carbon: Activated carbon is characterized by its high surface area, low manufacturing cost and ordered porous structure. It exhibits remarkable CO_2_ capture capacity in ambient conditions. The formation of activated carbon usually involved two steps: calcination and activation [13], which is far more convenient as compared to other materials. Recently, it has been found that many forms of activated carbon can be derived from waste biomass, such as palm leaf [14], rice husk [15], and cucumber peels [16]. Not only can these biomass-derived porous carbon forms effectively adsorb CO_2_, but they can also facilitate waste recycling. However, reliance on physisorption poses challenges in separating CO_2_ from mixed gases, and the material’s instability under practical flue gas conditions may impede its widespread application [17].

Metal oxide: Metal oxide can provide active sites that facilitate the adsorption and activation of CO_2_. Previous research found that: (1) the synergistic effect at the metal/oxide interface can regulate the adsorption and transformation of CO_2_; (2) the activity and selectivity of oxide-supported metal catalysts are governed by the binding strength of CO_2_ and intermediates at the metal/oxide interface; (3) by altering the binding strength at the metal/oxide interface, the pathway and selectivity of CO_2_ hydrogenation reactions can be modulated [18]. Metal oxides not only exhibit their own potential for converting CO_2_ into value-added long chain hydrocarbons [19,20], but they can also act as an effective promoter to boost the reaction activity by altering the electronic structure and providing electrons [21]. However, many metal oxides often exhibit low activity in reactions, necessitating the use of doping and other techniques to modulate their catalytic properties.

Metal-supported catalysts: Metal-supported catalysts typically exhibit superior particle morphology and mechanical strength, facilitating their separation from the reaction system and enabling recycling and reducing costs. So far there are various supports, including CeO_2_, ZrO_2_, and TiO_2_, that have been used in metal-supported systems. The type of support leads to the formation of different reaction intermediates, thereby significantly affecting the activity and selectivity of the catalyst [22]. In addition, various noble metals, including Ru and Pt, as well as non-noble metals, including Ni, Co, and Fe, are commonly utilized. However, some metals tend to form carbide phases during the reaction process. The formation of these phases increases the proportion of weakly adsorbed CO species, thereby enhancing the selectivity towards CO. Once the carbonaceous species are removed from the catalyst surface through oxidation, the catalyst can regain its selectivity for the original products [23]. This also raises an issue common to many metal-supported catalysts: the formation of carbon deposition and catalyst sintering during the reaction.

Graphene-based catalysts: Graphene-based catalysts exhibit superior performance in CO_2_ hydrogenation due to their intrinsic properties. These include exceptional chemical and thermal stability, superior thermal conductivity, and the capacity for nitrogen doping to generate basic sites that facilitate CO_2_ activation. Furthermore, the presence of the concentrated distribution of defects is beneficial for the dispersion and stabilization of metal or oxide nanoparticles. The application prospects of graphene-based catalysts in CO_2_ hydrogenation to methanol, formic acid, and olefin are promising [24]. However, under certain reaction conditions, graphene-based materials may lose their structural integrity or undergo chemical changes, affecting their long-term stability and catalytic performance.

Two-dimensional carbide-based catalysts: Mo_2_C is a typical material that, owing to its superior catalytic properties, is considered a promising candidate as a 2D carbide-based catalyst, particularly for the production of CO via the reverse water–gas shift (RWGS) reaction [25]. The 2D-Mo_2_C has demonstrated exceptional stability over more than 100 h of continuous operation, without any significant signs of deactivation. When compared to the industrially utilized Cu/ZnO/Al_2_O_3_ catalyst, 2D-Mo_2_C exhibits enhanced activity and stability under identical testing conditions. As a novel CO_2_ hydrogenation catalyst, 2D carbide-based catalysts not only boast high activity and selectivity but also possess excellent stability, which holds significant potential for industrial applications, though the 2D architecture may restrict the accessibility of active sites.

While these materials show potential for CO_2_ capture and conversion, each faces distinct challenges that necessitate further research and development to enable their practical deployment on an industrial scale [17,26,27,28].

The design principle of materials is intrinsically linked to the mechanisms of CO_2_ adsorption and reduction. The mechanism of CO_2_ adsorption can be broadly categorized into physisorption and chemisorption [29]. Physisorption involves weak Van der Waals force and electrostatic force between the adsorbent and adsorbate. This necessitates the utilization of materials endowed with high specific surface areas, well-defined porous structures, and suitable pore volumes and diameters. Conversely, chemisorption involves the formation of new substances through the interaction of the adsorbent with CO_2_. With respect to CO_2_ reduction, external energy inputs are essential to disrupt the stable molecular structure of CO_2_. CO_2_ reduction mainly yields new substances such as CO, CH_4_, CH_3_OH, and C_2_H_4_ [30], which hold industrial utility. Catalysis stands as a prevalent strategy for the CO_2_ reduction. The rational design of a catalyst can effectively promote the catalytic reaction. In general, desirable catalysts should feature uniformly dispersed and fully exposed active catalytic centers or, alternatively, exhibit high compatibility with other materials to augment their catalytic activity. 

In this regard, considering high porosity, tunable chemical structure and large surface areas, metal–organic frameworks (MOFs), constructed by organic linkers and metal ions or clusters, appear to be a promising material class for CO_2_ capture and conversion [31]. These distinctive features are not present in the aforementioned materials. Furthermore, due to flexible electronic structure and atomically dispersed active sites, MOFs hold great potential for catalytic reduction of CO_2_ [32]. Notably, the intrinsic properties of MOFs can be enhanced through the regulation of organic ligands or metal centers, optimization of pore spaces, immobilization of specific functional groups, and encapsulation of guest molecules [33], thereby endowing MOFs with diverse functionalities. In addition to pristine MOFs, MOF composites and derivatives have been explored to further enhance CO_2_ capture and conversion efficiency [34]. For example, Shang et al. [35] successfully synthesized CuBTC@Graphene Oxide (GO) (BTC = 1,3,5-benzenetricarboxylate) via a facile method for CO_2_ capture and achieved a high adsorption capacity of 8.9 mmol g^−1^ at 273 K, 1000 mbar. Qian et al. [36], using carbon dots (CDs) as a photosensitizer (PS) and Co(Hmim)_2_/In-BDC (ZIF-67/In-MIL-68, Hmim: 2-methylimidazole, BDC: 1,4-benzenedicarboxylate) as precursor, fabricated a ternary composite CDs-Co_3_O_4_/In_2_O_3_ for CO_2_ photocatalysis and obtained a high CO generation rate of 2.05 μmol h^−1^ g^−1^. An et al. [37] demonstrated the potential of using MOFs as a tunable platform for the design of catalysts with bimetallic centers, which exhibit high ethanol selectivity. These materials demonstrate improved efficiency in comparison to their parent MOFs.

**Scheme 1:**
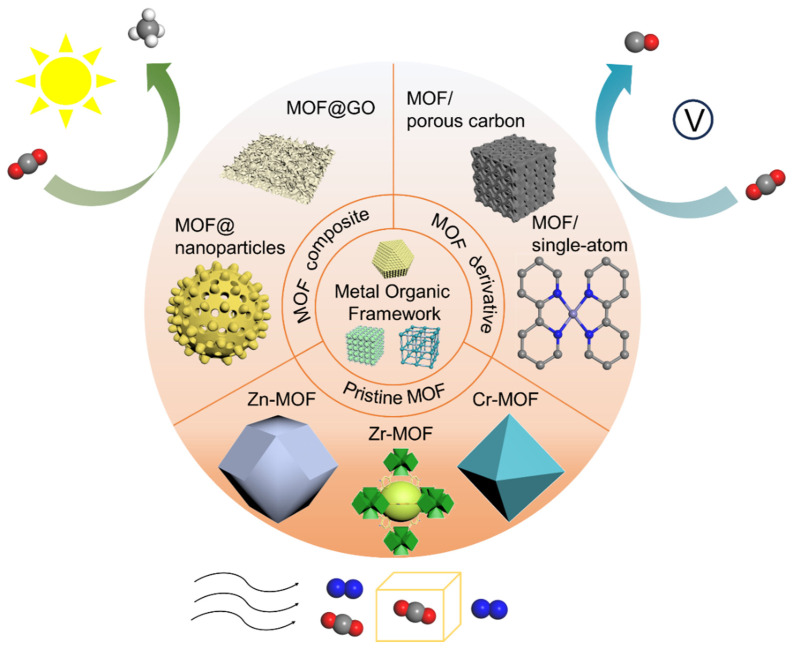
Schematic illustration of MOF-based materials (pristine MOF, MOF composites, and MOF derivatives) used in CO_2_ adsorption and photocatalytic and electrocatalytic reduction.

In fact, there have been many review articles discussing MOF-based materials for CO_2_ capture or conversion. As early as 2005, Millward et al. [38] first proposed MOFs as an efficient material for CO_2_ storage. In the last 15 years, in 2011, Bae et al. [39] emphasized the potential applications of MOFs in the field of CO_2_ separation and capture, and proposed criteria and strategies for the evaluation and design of novel MOFs. Just one year later, Hunger et al. [40] illustrated that when utilizing MOFs as additives in Mixed Matrix Membranes (MMMs), they demonstrate the potential to enhance CO_2_ gas separation performance. Particularly in the past 10 years, there have been more and more research studies focusing on MOFs for CO_2_ capture and utilization. For instance, in 2014, Alhamami et al. [41] primarily discussed the “breathing” behavior of MOFs in the context of CO_2_ adsorption, with an emphasis on the potential spatial framework requirements necessary for the manifestation of breathing behavior in MOFs. In 2015, Kathalikkattil et al. [42] pointed out that MOFs can serve as effective materials for catalyzing the chemical fixation of CO_2_ through cycloaddition with epoxides to produce cyclic carbonates and discussed strategies for enhancing the performance of MOF catalysts, as well as the challenges and prospects for realizing the industrial application of these catalysts. In 2016, He et al. [32] reported the application of MOFs in CO_2_ chemical transformation, including photo and electroreduction, chemical fixation of CO_2_ with epoxides, and CO_2_ chemically fixed onto MOFs or terminal alkynes. Three years later, Ding et al. [43] concluded the recent progress and provided an account of the design and synthesis of MOF-based materials used in CO_2_ capture and conversion. In 2020, focused on the CO_2_ adsorption, Ghanbari et al. [31] discussed the adsorption properties and mechanism of CO_2_ on MOF and the design of MOF structures. In 2021, Zhao et al. [44] summarized some MOF-based materials, including pristine MOFs, MOF hybrids, and MOF-derived carbon-based single-atom catalysts (SACs) used in CO_2_ electrocatalytic reduction. Most recently, in 2022 and 2024, Fan et al. [45] provided an insight into cobalt-based MOF photocatalysts for CO_2_ reduction to renewable energy and Liu et al. [46] discussed carbon-neutral catalysis based on MOFs in terms of opportunities and challenges. This article reviews the recent progress of CO_2_ capture and conversion by using MOF-based materials. Processes and fundamentals of MOF-based materials for CO_2_ adsorptive separation, photocatalytic and electrocatalytic reduction, CO_2_ hydrogenation reduction, and dual functionalities are elaborated. Moreover, different strategies for enhancing MOF-based materials’ capacity, including modification of organic ligands and metal centers, fabrication of MOF composites, and MOF derivatives are summarized (Scheme 1). This work aims to foster a deep understanding of the recent progress in MOF-based materials in CO_2_ removal and provides insights for future research.

## 2. MOFs for CO_2_ Capture and Conversion

CO_2_ capture can be approached through different methods, such as direct air capture (DAC) or point-source capture, each characterized by distinct CO_2_ concentration levels. Therefore, the design of MOFs should be tailored to accommodate the specific concentrations of CO_2_ encountered in these varying scenarios. This requires MOFs to be adaptable and efficient across a range of CO_2_ concentrations, from the relatively low levels found in ambient air to the higher concentrations emitted by industrial processes. Under conditions of low concentration of CO_2_, MOF design should be inclined to high specific surface area to maximize the interaction between MOF and CO_2_, to reasonable aperture to selectively adsorb CO_2_ molecules, and to strong CO_2_-philic ligands to enhance the interaction with CO_2_. In addition, the stability and regenerability of MOFs under actual DAC conditions are crucial, particularly for the effects of humidity and temperature [47]. Under conditions of high concentration of CO_2_, ensuring that MOF exhibits fast adsorption and desorption rates, maintains its structural integrity and performance under the harsh conditions, and shows a good resistance to coking are critical design strategies. The following are some regulation strategies of MOFs.

### 2.1. Tunable Chemical Structure

#### 2.1.1. Open Metal Sites (OMSs)

**Figure 2:**
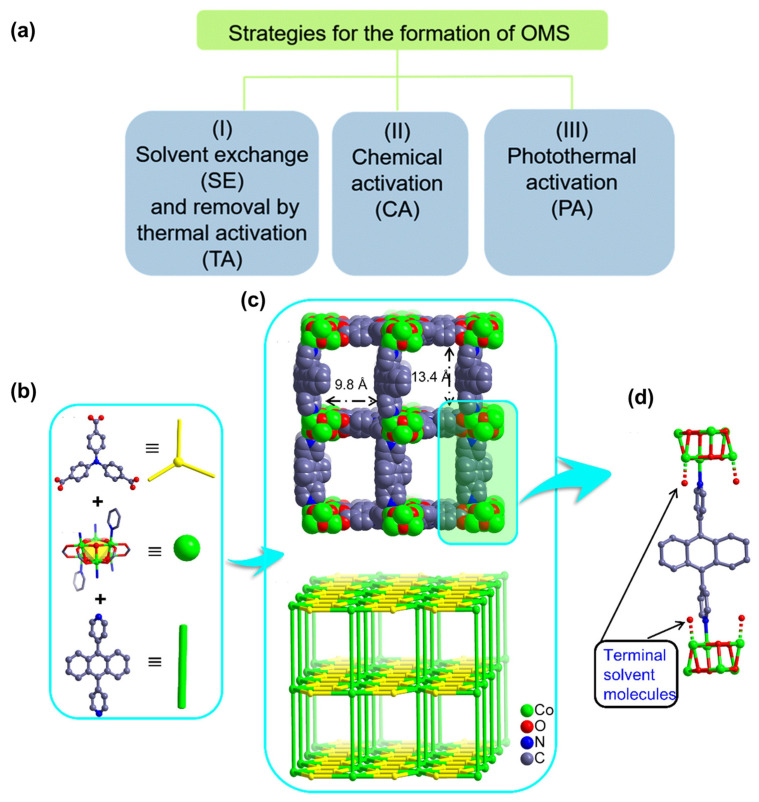
(**a**) The generation of OMSs through different methods. Reproduced from [48]. Copyright 2020, Royal Society of Chemistry. (**b**) The structure of TCA^3−^ ligand (top), Co_6_(µ_3_-OH)_6_ cluster (middle), and ABPY ligand (bottom). (**c**) The structure (top) and topology of DZU-6 (bottom). (**d**) Link mode between ABPY ligand and [Co_6_(µ_3_-OH)_6_] clusters with terminal solvent molecules. Reproduced from [49]. Copyright 2022, Elsevier.

The development of OMSs with unsaturated metal coordination environments represents a prominent strategy for CO_2_ capture. In typical MOF synthesis, metal centers are frequently coordinated by terminal ligands, which occupy the coordination sphere around the metal ions. However, these molecules can be removed through subsequent heating and activation, resulting in the formation of OMSs (Figure 2a). Importantly, the activation process must ensure the preservation of the original framework’s integrity. It is imperative that the inherent crystallinity and porosity of the material are maintained [48]. 

Accordingly, OMSs function as Lewis acid sites, capable of binding CO_2_ molecules through electrostatic interaction. In a representative example [49], accessible open Co sites were engineered by removing terminal solvent molecules in a pillar-layered MOF based on [Co_6_(µ_3_-OH)_6_] clusters linked by TCA^3−^ (4,4′,4″-tricarboxyltriphenylamine) and ABPY (4,4′-(9,10-anthracenediyl)bis-Pyridine) ligands (designated as DZU-6). The structure and topology of DZU-6 is shown in Figure 2b–d. Following activation, the structural integrity of DZU-6 is confirmed via thermogravimetric analysis (TGA) and N_2_ adsorption measurements, indicating no loss of thermal stability and porosity. Subsequent CO_2_ adsorption/desorption isotherms and isosteric heat of CO_2_ adsorption (Q_st_) analyses demonstrated an over 40% increase in CO_2_ capacity and enhanced interaction with CO_2_ molecules in DZU-6 compared to its non-OMS counterpart. The above results underscore the pivotal role of OMSs in MOFs, which confer a strong affinity for trapping CO_2_ molecules.

#### 2.1.2. Ligand Modification

It is important to note that the adsorption mechanism of OMSs is primarily governed by physisorption, a process that is inherently weak and less effective under low-pressure conditions. To surmount these limitations, the introduction of functional groups via ligand modification emerges as a viable strategy. Functionalized MOFs can be obtained through either pre-synthesis or post-synthesis modifications. Pre-synthesis modification entails the incorporation of organic ligands bearing functional groups at the onset of MOF synthesis. Conversely, post-synthesis modification involves the inclusion of functional groups into the as-synthesized MOFs. 

**Figure 3:**
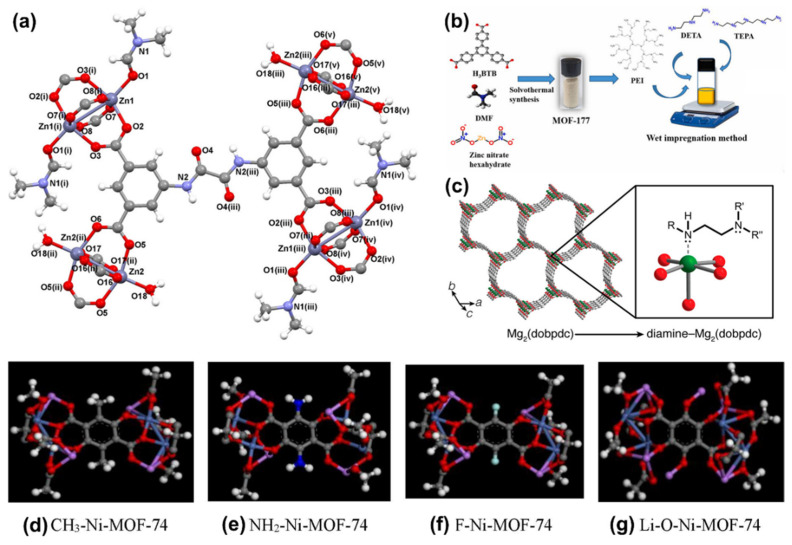
Illustration of (**a**) {[Zn_3_(μ_8_-OATA)_1.5_(H_2_O)_2_(DMF)]·5/2H_2_O·5DMF}*_n_* (Zn-OATA) functionalized by oxalamide groups. H_4_OATA: N,N′-bis(3,5-dicarboxyphenyl)oxalamide. Reproduced from [51]. Copyright 2021, American Chemical Society. (**b**) Synthetic procedure and functionalization of Zn_4_O(BTB)_2_ (MOF-177) by polyethyleneimine (PEI), tetraethylenepentamine (TEPA), and diethylenetriamine (DETA). Reproduced from [52]. Copyright 2021, Elsevier. (**c**) Illustration of Mg_2_(dobpdc) (dobpdc^4–^ = 4,4′-dioxidobiphenyl-3,3′-dicarboxylate) functionalized by diamine groups. Reproduced from [53]. Copyright 2021, American Chemical Society. Illustration of Ni-MOF-74 functionalized by various functional groups. (**d**) Ni-MOF-74 functionalized by –CH_3_ groups. (**e**) Ni-MOF-74 functionalized by –NH_2_ groups. (**f**) Ni-MOF-74 functionalized by –F groups. (**g**) Ni-MOF-74 functionalized by –Li-O groups. MOF-74 consisted of transition metal cations (M) (M = Zn^2+^, Mg^2+^, Co^2+^, Cd^2+^, Mn^2+^, Fe^2+^, or Ni^2+^) and the dobdc^4−^ or dhtp^4−^ (2,5-dihydroxyterephthalate) ligand. (C: gray; O: red; H: white; N: blue; F: green; Li: pink) Reproduced from [55]. Copyright 2021, Elsevier.

Among the various functional groups, amine groups exhibit exceptional potential owing to their cost-effectiveness, high amine density, favorable absorption heat, and facile preparation. Additionally, they also demonstrate a robust CO_2_ absorption capacity, particularly under ultra-dilute conditions [50]. The incorporation of amine groups is instrumental in creating Lewis basic sites (LBSs), enhancing the chemisorption ability of CO_2_ through Lewis acid-base interactions. It has been shown that the presence of LBSs significantly reinforces the binding affinity between CO_2_ and MOFs. To date, numerous amine precursors, including amides [51] (Figure 3a), polyamines [52] (Figure 3b), and diamines [53] (Figure 3c), have been utilized to modify MOFs, resulting in amine-functionalized MOFs with heightened CO_2_ binding energy.

Beyond amine groups, a variety of other functional groups, including –CH_3_, -F, and -O-Li, can also be employed for the modification of MOFs (Figure 3d–g). Certain functional groups contribute to enhancing the materials’ hydrophobicity, thereby improving the CO_2_ separation performance under humid conditions [43,54,55].

#### 2.1.3. Pore Size Control

Undoubtedly, the inborn high porosity and diverse pore sizes of MOFs play an important role in CO_2_ capture. Given that the molecular kinetic diameter of CO_2_ molecules is estimated to be 3.3 Å, macropores and mesopores are not appropriate for confining CO_2_ within the channels. Thus, precise regulation of pore dimensions to micropores becomes significant. In essence, adjusting the pore size to align with the molecular dimensions of the target gas enhances the sieving effect [56], where large molecules are excluded due to pore size restrictions, while small molecules are accommodated. 

**Figure 4:**
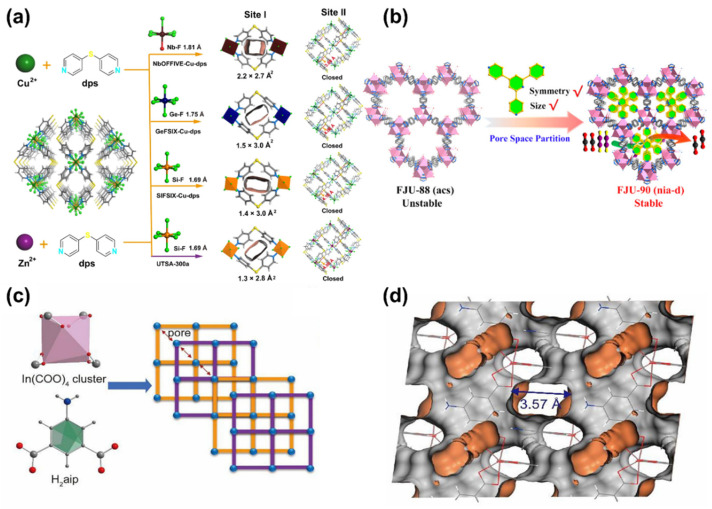
(**a**) Synthetic procedure of NbOFFIVE-Cu-dps, GeFSIX-Cu-dps, SIFSIX-Cu-dps, and UTSA-300a, and their corresponding pore aperture size in site I and II. Color code: Cu, green; F, light green; S, bright yellow; N, light blue; C, gray; Si, orange; Ge, navy blue; Nb, wine red; solvent molecules are omitted for clarity. Reproduced from [58]. Copyright 2022, Springer Nature. (**b**) Pore space partition strategy for FJU-88 through ligand insertion. Reproduced from [61]. Copyright 2019, American Chemical Society. (**c**) Synthetic procedure of In(aip)_2_ and the corresponding 2D layers along *c* axis (corner: In cluster; edge: ligand). (**d**) The schematic illustration of pore structure of In(aip)_2_ along *b* axis. Reproduced from [59]. Copyright 2022, Elsevier.

One strategy for controlling the pore size is the rational selection of coordination networks of secondary building units (SBUs). SBUs are fundamental to the architectural diversity of MOF topologies [57]. The topology determines the resulting pore size in MOFs. Through combining rigid building units of varying geometry and multibranched organic linkers, MOFs with diverse pore structures can be synthesized [56]. For example, the CO_2_/C_2_H_2_ separation performance can be modulated by altering anionic linkers to form a series of SIFSIX-dps-Zn variants, SIFSIX-dps-Cu (SIFSIX  =  SiF_6_^2−^, dps  =  4.4′-dipyridylsulfide), GeFSIX-dps-Cu (GeFSIX  =  GeF_6_^2−^), and NbOFFIVE-dps-Cu (NbOFFIVE  =  NbOF_5_^2−^), as a consequence of suitable pore size adjustment (Figure 4a) [58]. Similarly, the pore size in an In-based MOF is composed of 5-aminoisophthalic (aip) ligand and In(III) cluster; In(aip)_2_) can be precisely controlled within the range of 3.4~3.6 Å (Figure 4c,d). Such exclusive pore size is responsible for efficient CO_2_ sieving [59]. 

Pore space partition (PSP) is another strategy developed by Zhai’s group in 2017 [60]. This concept involves dividing the original cage or channel space into smaller segments by inserting a proper partition agent, such as additional organic linkers or guest species. Guided by the PSP concept, Ye et al. [61] immobilized a triangular ligand (Tripp = 2,4,6-tris(4-pyridyl)pyridine) into the cage of a porous MOF ([Co_3_(μ_3_-OH)(CPT)_3_(H_2_O)_3_]Cl_2_(DMA)_5.5_(H_2_O)_5_, FJU-88) to achieve space partition (Figure 4b). Correspondingly, the pore apertures in the partitioned MOF were reduced from 12.0 × 9.4 to 5.4 × 5.1 Å^2^. The narrowed pore sizes were favorable for separating CO_2_ and C_2_H_2_.

### 2.2. Precursors and Templates

**Figure 5:**
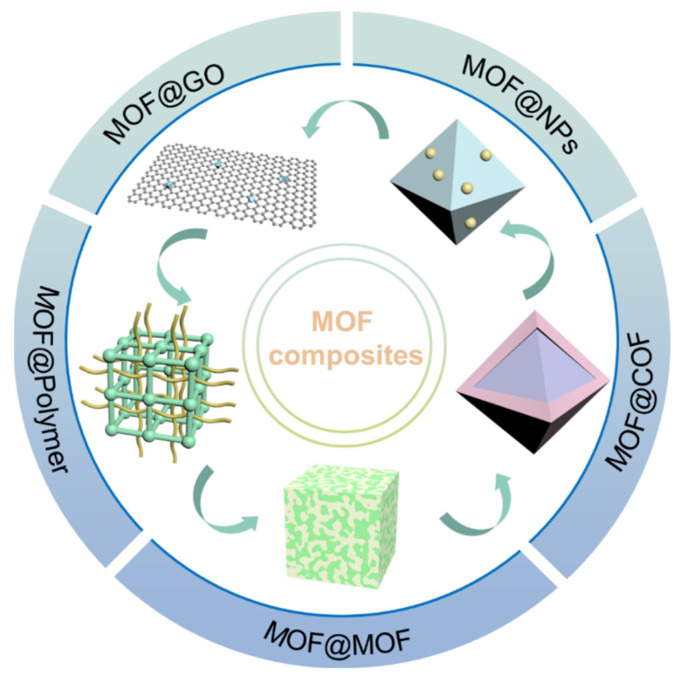
Design strategies of MOF composites.

The integration of other materials with MOFs to fabricate MOF composites or MOF derivatives represents a useful design strategy. In such cases, the resulting compounds not only inherit the advantages of the original MOFs but also introduce new benefits, including new pore environments [43], larger surface areas, and additional active sites. Numerous materials, including nanoparticles (NPs), covalent organic frameworks (COFs), polymers, and GO, have been recognized for their synergistic interactions when hybridized with MOFs (Figure 5) [62]. Likewise, MOF-derived porous carbon [63], metal compounds [64], and SACs [65] also have a promising application prospect in CO_2_ conversion. Needless to say, MOF composites and derivatives significantly enhance CO_2_ adsorption and catalysis activity, surpassing their parent MOFs in both CO_2_ capture and conversion. A variety of MOF composites will be discussed in detail in the following passage.

## 3. CO_2_ Capture, Separation, and Conversion

### 3.1. CO_2_ Capture and Separation

Carbon capture emerges as a crucial component of CCU technology, acting as both an initial part and a key process. Following capture, CO_2_ also has to be separated from the gas mixture. During the capture phase, CO_2_ is extracted from the atmosphere or industrial plant emissions and absorbed by adsorbents. Currently, carbon capture technologies can be divided into three main categories: pre-combustion capture, oxy-combustion capture, and post-combustion capture [66,67,68]. Research indicates that post-combustion capture holds the greatest potential in the application of adsorbents [69,70]. In this process, as the gas mixture passes through adsorbents, CO_2_ is selectively captured while other gases are discharged with the flue gas, thus achieving CO_2_ separation. 

**Figure 6:**
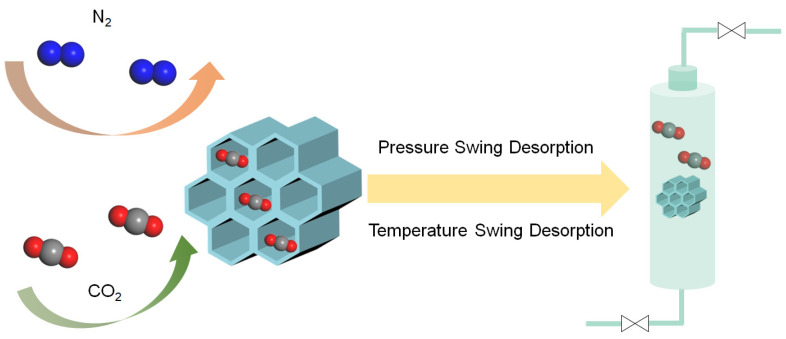
Schematic illustration of CO_2_ capture and separation.

Subsequent to the capture phase, the captured CO_2_ must be released from the adsorbents, a process known as desorption (Figure 6). Typically, CO_2_ desorption is achieved through variations in temperature or pressure [31]. Increasing the temperature or lowering the pressure induces the gradual detachment of CO_2_ from the adsorbents, leading to its enrichment. Desorption is an essential process for enabling further CO_2_ utilization and facilitating adsorbent regeneration.

### 3.2. CO_2_ Conversion

In terms of sustainable development, both light and electricity are recognized as clean and sustainable energy sources. Photocatalysis and electrocatalysis based on light and electricity are considered as straightforward pathways for CO_2_ conversion. These processes necessitate the involvement of electrons and protons in the reduction of CO_2_. Notably, the resultant reduction products can be categorized based on the number of electrons acquired during the reaction: (1) two-electron products HCOOH and CO; (2) four-electron product HCHO; (3) six-electron product CH_3_OH; (4) eight-electron product CH_4_; (5) twelve-electron product C_2_H_4_. The specific equations are shown in Table 1.

#### 3.2.1. CO_2_ Photocatalytic Reduction

**Figure 7:**
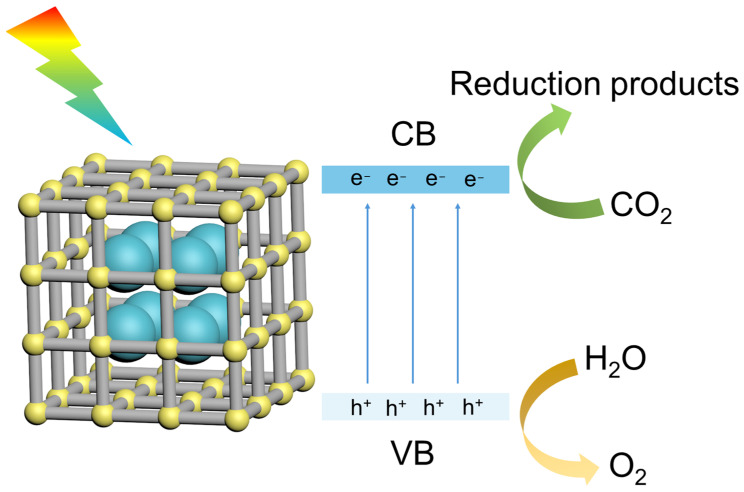
The mechanism of photocatalytic reduction of CO_2_.

The photocatalytic reduction of CO_2_ hinges upon the photoconductive properties of the materials. Upon absorption of photons, electrons initially situated in the valence band (VB) become excited and transfer to the conduction band (CB), leaving behind holes in their original positions. These photo-induced electrons possess the capacity to drive CO_2_ reduction, whereas the corresponding photo-induced holes can oxidize water to produce oxygen [71] (Figure 7). To facilitate the requisite redox reactions, several conditions must be met: (1) the energy of the incident photon must exceed the band gap of the material; (2) the potential of the CB edge should be more negative than the CO_2_ redox potential and the potential of the VB edge should be more positive than the water redox potential. In particular, since the thermodynamic potential of water oxidation to yield H_2_ and O_2_ is more favorable compared to CO_2_ reduction, a large amount of energy should be input to overcome the thermodynamic and kinetic barriers of CO_2_ reduction [72]. Crucially, the effective separation of photo-induced electron–hole pairs is the pivotal factor in determining the efficacy of CO_2_ photocatalytic reactions; failure to achieve adequate separation may lead to rapid recombination of photo-induced carriers.

#### 3.2.2. CO_2_ Electrocatalytic Reduction

The fundamentals of electrocatalytic reduction encompass a series of intermediates, with the adsorption and desorption of key intermediates exerting significant influence on the reactions. 

**Figure 8:**
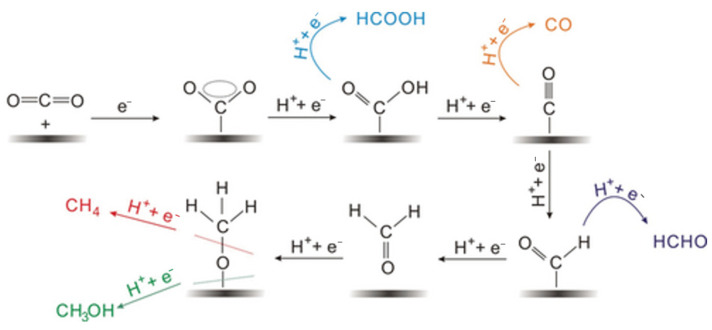
Possible reaction pathways for electrocatalytic reduction of CO_2_ to C1 products. Reproduced from [73]. Copyright 2017, Wiley.

Upon adsorption of the CO_2_ molecule onto the catalyst surface, large negative potentials should be applied to initially break the stable linear geometry for CO_2_, a process known as activation. It is widely accepted that the initial step in CO_2_ electrocatalytic reduction involves the acquisition of one electron, resulting in the formation of active phase *CO_2_^−^. Subsequently, the activated CO_2_ molecules proceed to accept electrons and protons, leading to the formation of various intermediates. C1 products represent the most easily generated products in CO_2_ electrocatalytic reactions. The formation of HCOOH involves the CO_2_^−^ accepting a proton (H^+^) to form either bicarbonate (HCO_3_^−^) or formate (HCOO^−^), and then undergoes further reduction to form the key intermediate *OCHO, which can be further converted into HCOOH. Alternatively, CO_2_ may directly transform into the *COOH intermediate, which subsequently accepts a proton to form HCOOH. On another pathway, the HCO_3_^−^ can further accept an electron and a proton to form *COOH. This *COOH intermediate then undergoes a transformation by losing an oxygen atom and gaining two electrons to become *CO. Then, *CO desorbs from the catalyst surface, releasing it as gaseous CO. The H_3_CO intermediate plays a crucial role in the formation of both CH_3_OH and CH_4_, as it can undergo a series of electron and proton transfer reactions to be transformed into either CH_3_OH or CH_4_ (Figure 8). Moreover, the C–C coupling reaction results in the formation of various C2 products [44,73].

#### 3.2.3. CO_2_ Hydrogenation

Hydrogen, as a green and renewable energy vector, has emerged as a compelling alternative to petroleum-based energy sources. The generation of H_2_ through the electrolysis of water presents a dual solution to the ecological and energy crises. CO_2_ hydrogenation in the presence of green H_2_ is thus recognized as a sustainable and viable route for the synthesis of high-value fuels [74].

In this article, we primarily discuss CO_2_ hydrogenation to produce C1 products. CO_2_ hydrogenation to C1 products can be grouped into three main categories: RWGS reaction; CO_2_ methanation; CO_2_ methanolation [30]. The equations are as follows:

RWGS reaction: CO_2_ + H_2_ = CO + H_2_O ΔH_298K_ = 41.2 kJ mol^−1^

CO_2_ methanation: CO_2_ + 4H_2_ = CH_4_ + 2H_2_O ΔH_298K_ = −165.1 kJ mol^−1^

CO_2_ methanolation: CO_2_ + 3H_2_ = CH_3_OH + H_2_O ΔH_298K_ = −49.5 kJ mol^−1^

The RWGS reaction is thermodynamically favored at high temperatures due to its endothermic nature. The resultant syngas serves as a key intermediate for various chemical syntheses. Specifically, it can be utilized in the synthesis of ammonia, methanol, and through processes such as hydroformylation and the Fischer–Tropsch reaction, for the production of liquid hydrocarbons [75]. 

The Sabatier reaction, involving CO_2_ hydrogenation to CH_4_, is a critical pathway for converting CO_2_ into a valuable energy carrier. The resultant CH_4_ is compatible with existing natural gas infrastructure, allowing for direct pipeline injection or utilization as a fuel and chemical feedstock. Although the Sabatier reaction is exothermic and thermodynamically favorable, it faces kinetic challenges due to the requirement for an eight-electron reduction to produce CH_4_. However, with the development of appropriate catalysts, this reaction can be effectively conducted at atmospheric pressure, offering a milder alternative to high-pressure processes [76]. In addition, it is also feasible to operate CO_2_ methanation at a low temperature (below 300 °C) [77,78], which helps to mitigate the risk of catalyst deactivation through sintering and agglomeration, and also prevents the occurrence of the RWGS reaction. Consequently, CO_2_ methanation is considered advantageous for CO_2_ hydrogenation due to its favorable thermodynamics and the potential for operation under mild conditions.

Methanol finds extensive usage in various applications, including organic synthesis, pesticides, medicine, and as an energy carrier, positioning it as a promising substitute for conventional fossil fuels [79]. The synthesis of methanol can be achieved either through direct CO_2_ hydrogenation or via CO hydrogenation. However, the industrial synthesis of methanol predominantly employs syngas due to the more favorable thermodynamic and kinetic properties of CO in comparison to CO_2_ [80]. Overcoming the thermodynamic and kinetic barrier remains a challenge in the direct synthesis of methanol from CO_2_ hydrogenation. Thermodynamically, the synthesis of methanol from CO_2_ is optimally conducted at low temperatures and high pressures to minimize the generation of CO as a byproduct [79,81]. Importantly, recent research has validated the feasibility of methanol production from CO_2_ at room temperature, which represents a notable advancement in the utilization of CO_2_ [82]. 

## 4. MOF-Based Materials for CO_2_ Capture and Separation

Capture (adsorption) stands out as one of the most outstanding properties of MOFs. Structurally, MOFs boast a high specific surface area and a porous framework, capable of accommodating adsorbate molecules. Chemically, the pore size and surface functional groups can be precisely controlled through the meticulous manipulation of metal centers or organic ligands, ensuring compatibility with target adsorbate molecules. In addition, MOFs serve as exceptional substrates for the incorporation of other materials, leading to synergistic effects between the two components. In this section, we will delve into pristine MOFs, structural modulation of MOFs, and MOF composites. The performances of various MOF-based materials, including pristine MOFs and MOF composites are illustrated in Table 2 and Table 3, respectively.

### 4.1. Pristine MOFs

**Figure 9:**
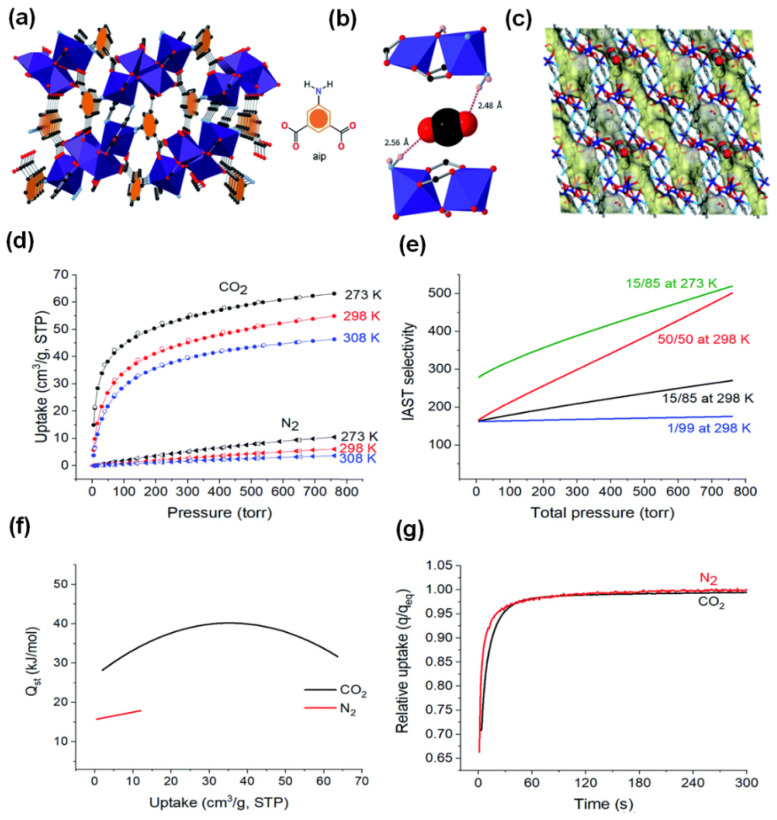
(**a**) The structure of MUF-17 with pentanuclear Co(II) clusters connected by twelve dianionic aip linkers (cobalt: dark blue; oxygen: red; carbon: dark grey; nitrogen: light blue; hydrogen atoms are omitted for clarity). (**b**) CO_2_ binding through hydrogen bonding, (calculated by DFT). (**c**) The yellow and grey Connolly surface (1.0 diameter probe) shows the one-dimensional pore network of MUF-17 and the position of the CO_2_ molecules at the narrowest channel neck. (**d**) CO_2_ (circle) and N_2_ (triangle), uptake adsorption (solid symbols), desorption (open symbols), isotherms at different temperatures. (**e**) Q_st_ plots for CO_2_ and N_2_. (**f**) The IAST selectivities of CO_2_/N_2_ with different proportions at 298 K and 273 K. (**g**) The kinetic profile of CO_2_ and N_2_ absorption of MUF-17 at 293 K after exposure to a gas dose equal to the total adsorption of the gas measured at 1 bar in the evacuated sample; q refers to the amount of absorption at time t, q_eq_ refers to the equilibrium rate of absorption at 293 K, 1 bar. Reproduced from [83]. Copyright 2020, Royal Society of Chemistry.

An ideal MOF for practical industrial production must have the following properties: easy and cost-effective synthesis procedure, high CO_2_ adsorption and separation performance, good stability, sustainability, mild regeneration energy consumption, and the ability to produce on a large scale. In 2020, a robust and novel MOF, dubbed MUF-17 ([Co_5_(μ_3_-OH)_2_(aip)_4_(H_2_O)_2_]) (MUF = Massey University Framework) was prepared by Qazvini’s groups [83]. They used cobalt acetate and 5-aminoisophthalic acid as the starting materials due to their cost-effectiveness. A reflux method instead of the conventional energy-intensive solvothermal synthesis was used as a greener synthesis approach. The as-prepared material featured a pentanuclear Co(II) cluster connected by twelve dianionic aip linkers (Figure 9a). MUF-17 showcases narrow zigzag one-dimensional pores, with cavities that have apertures around 4.7 × 4.8 Å, interconnected by narrow channels of approximately 3.1 × 3.5 Å (Figure 9c). Powder X-ray diffraction (PXRD) and gas isothermal analysis affirm its exceptional crystallinity, high purity, and thermal stability up to 280 °C, even in the presence of water vapor and oxygen. Notably, MUF-17 exhibits remarkable CO_2_ capture capability at low concentrations (55.0 cm^3^ g^−1^) with negligible N_2_ uptake (6.1 cm^3^ g^−1^) at 1 bar and 298 K (Figure 9d). This is attributed to its desirable electrostatic and hydrogen bonding interactions with CO_2_, as evidenced by density functional theory (DFT) calculations (Figure 9b). Ideal adsorbed solution theory (IAST) highlights its impressive selectivity toward CO_2_ over N_2_ (Figure 9f). Furthermore, the calculated Q_st_ for CO_2_ is 28.3 kJ mol^−1^ (Figure 9e), indicating favorable gas binding strength and ease of regeneration. In spite of impressive adsorption capacity and selectivity, MUF-17 also demonstrates rapid adsorption kinetics for both CO_2_ and N_2_ at 293 K, reaching equilibrium capacity in less than 60 s (Figure 9g). This fast kinetics is advantageous for the adsorptive separation process under dynamic conditions. Subsequent breakthrough experiments with varying CO_2_/N_2_ gas mixtures confirmed the high efficiency of MUF-17 in dynamic CO_2_/N_2_ separation. Impressively, MUF-17 exhibits outstanding recyclability for at least 30 cycles and maintains efficient separation performance even in the presence of water.

**Figure 10:**
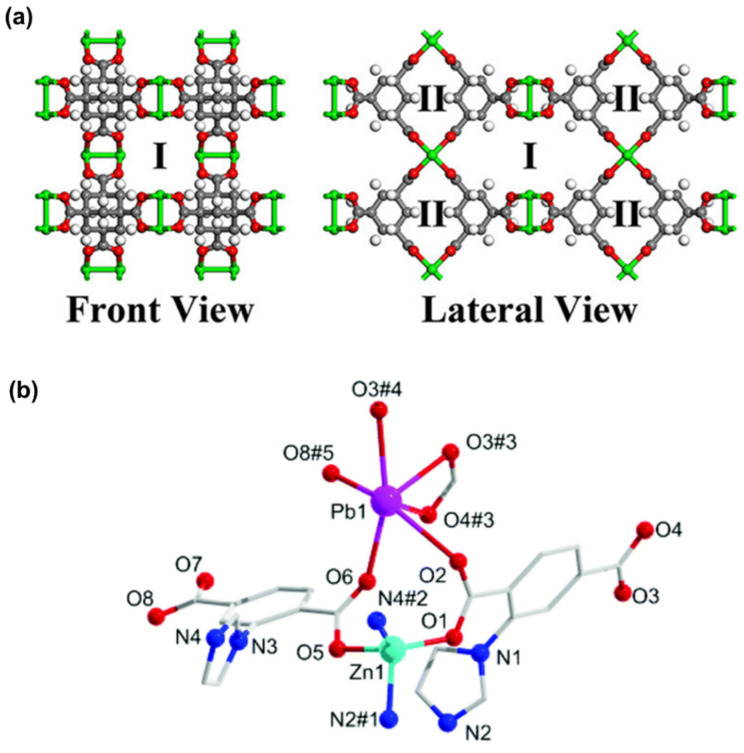
(**a**) Structure illustration of MOF-11 with channel Ι and channel II. (Cu: green; O: red; C: gray; H: white). Reproduced from [85]. Copyright 2021, American Chemical Society. (**b**) Coordination environment of HMOF with unsaturated Pb(II) and Zn(II) sites. Symmetry codes for HMOF: #1, 1 − *x*, 1 − *y*, 1 − *z*; #2, −*x*, 2 − *y*, 1 − *z*; #3, 1 − *x*, 1 − *y*, −*z*; #4, −1 + *x*, −*y*, −*z*; #5, −*x*, 1 − *y*, 1 − *z*. Reproduced from [86]. Copyright 2020, Royal Society of Chemistry.

OMSs, functioning as Lewis acid sites, significantly enhance CO_2_ affinity through electrostatic interactions. An exemplary instance is MOF-11 (Cu_2_(ATC)·6H_2_O, ATC: 1,3,5,7-Adamantane Tetracarboxylate) [84], specifically engineered for CO_2_ adsorption and separation [85]. MOF-11 comprises two distinct chemical structures: one featuring dense Cu OMSs (channel Ι) and the other characterized by narrow pore window size (channel II) (Figure 10a). MOF-11 exhibits a stronger CO_2_ uptake at low pressure, mainly attributed to the dense Cu OMSs. In channel Ι, multiple O-Cu electrostatic interactions between CO_2_ molecules and Cu OMSs with high binding energy are observed, indicating preferential CO_2_ binding. Weak N^…^H-C Van der Waals interactions between N_2_ molecules and CH_2_ moieties in channel II suggest that N_2_ molecules tend to be absorbed into the window of pores. This unique pore architecture endows the framework with a robust CO_2_ affinity while simultaneously attenuating the N_2_ adsorption, thus achieving high CO_2_/N_2_ separation performance. This research underscores the promise of monometallic MOFs with OMSs in CO_2_ adsorption and separation.

Similarly, heterometallic MOFs (HMOFs) with OMSs exhibit excellent gas adsorption properties. An HMOF was prepared via Zn(II) ions and MOF ({[Pb_2_(L)_2_(H_2_O)]H_2_O}_n_, with 2-(imidazol-1-yl)terephthalic acid (H_2_L)) as precursors, yielding the chemical formula {[PbZn(L)_2_]·DMA·H_2_O}_n_ [86]. Upon removal of guest molecules, unsaturated Pb(II) and Zn(II) active sites, along with uncoordinated carboxylate oxygen atoms, are generated at the pore surface (Figure 10b). The activated HMOF is confirmed to possess a higher CO_2_ adsorption capacity of 39.3 cm^3^ g^−1^ (7.8 wt%) at 273 K and 25.5 cm^3^ g^−1^ (5.0 wt%) at 298 K compared to monometallic Zn(II)- and Pb(II)-based MOFs. Moreover, attributed to the synergistic effect between OMSs and uncoordinated carboxylate oxygen atoms, the activated HMOF also affords a remarkable CO_2_/CH_4_ selectivity, ranging from 4.5 to 16.3 at 298 K and 8.6 to 41.3 at 273 K for the equimolar gas mixtures. In short, constructing MOFs with OMSs is a useful and effective strategy to enhance affinity for CO_2_, since this is readily achievable through simple activation.

**Figure 11:**
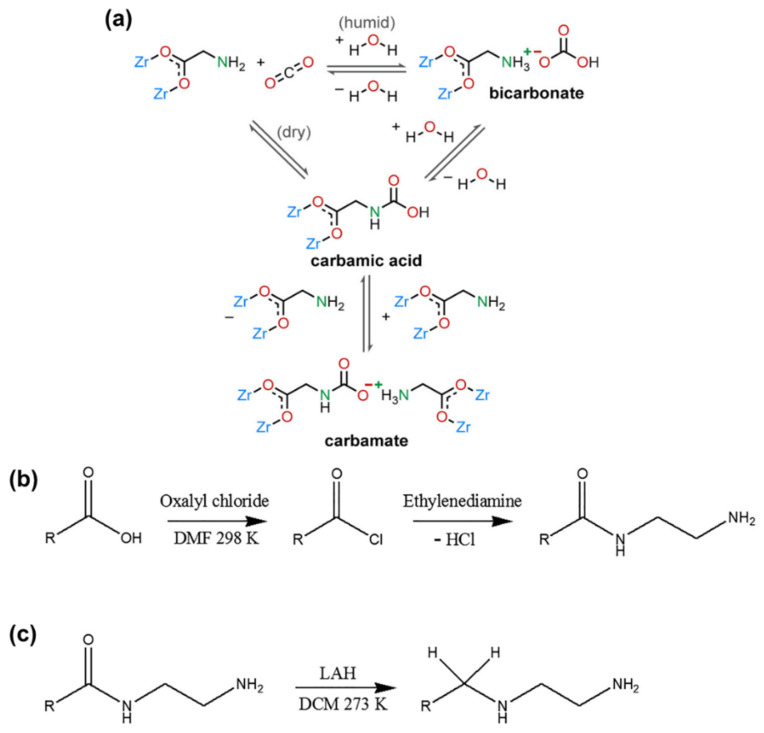
(**a**) Proposed mechanism of CO_2_ adsorption of MOF-808-AAs under both dry and humid conditions. Reproduced from [87]. Copyright 2022, American Chemical Society. Reaction schemes of (**b**) conversion of M808-EDTA into M808-EDTA-ED. (**c**) Conversion of M808-EDTA-ED into M808-EDTA-ED-R. Reproduced from [89]. Copyright 2020, Elsevier.

Amino-functionalized ligands exhibit exceptional CO_2_ adsorption capacity due to the Lewis basic-acid interaction. However, some amino groups may not withstand harsh conditions and compete with water molecules, leading to amino loss and diminished adsorption efficiency. Addressing this challenge, a robust MOF-808 [Zr_6_O_4_(OH)_4_-(BTC)_2_(HCOO)_6_] was synthesized and functionalized with a series of amino acids to create a humidity-enhanced CO_2_ uptake and low-energy regeneration material, termed MOF-808-AAs. In MOF-808-AAs, amino acid ligands are bound through carboxylation to the Zr(IV) center, forming a strong Zr(IV)-carboxylate interaction [87]. The amino groups (-NH_2_ or -NH) on alkyl chains are oriented toward pores as the main place to interact and capture CO_2_ from outside. Three mechanistic processes of CO_2_ adsorption in MOF-808-AAs are identified: (1) Formation of carbamates or carbamic acids under dry conditions; (2) Formation of carbamates or carbamic acids with higher affinity for CO_2_ in low pressures under humid conditions; (3) Formation of bicarbonates at high CO_2_ concentration under humid conditions (Figure 11a). Under dry conditions, the amount of CO_2_ uptake is relatively low, but upon the addition of water, CO_2_ uptake increases over twofold at 4 kPa, indicating enhanced CO_2_ affinity in the presence of water. The stoichiometry of bicarbonate formation under rich CO_2_ and wet conditions partly explains the improved CO_2_ capture performance of MOF-808-AAs. Notably, in the circumstance of (1), heat is required to regenerate the adsorbents. In contrast, an energy-efficient vacuum-swing process can be employed in the case of (3) without the need for heat input. The special pore environment, water-enhanced ability, and easy vacuum regenerability position MOF-808-AA as a promising candidate for CO_2_ capture under humid conditions. Similarly, a robust metal–triazolate framework appended by amino groups achieves CO_2_/H_2_O kinetic adsorption selectivity up to 70 and a 20% enhancement of CO_2_ capture in CO_2_/N_2_ breakthrough curves under humid conditions, along with strong endurance against various environmental conditions [88].

Amides are the products of a reaction between a carboxylic acid and ammonia or an amine. However, amide groups are found to be less effective than amino groups in CO_2_ adsorption. A representative example is MOF-808, which was first modified with ethylenediaminetetraacetic acid (EDTA), followed by reaction with ethylenediamine (ED) (Figure 11b), and finally reduced with lithium aluminum hydride (LAH) (Figure 11c) to form a series of materials: M808-EDTA, M808-EDTA-ED, and M808-EDTA-ED-R, respectively [89]. Among these, M808-EDTA-ED exhibits poor porosity, CO_2_ adsorption quantity, isosteric heat, and CO_2_ selectivity due to the high concentration of amide groups, resulting in low porosity and limited basic sites. However, further treatment with LAH converts ineffective amide groups into amino groups. The occurrence of amino groups extensively contributes to the increase in basic sites. As observed, M808-EDTA-ED-R demonstrates the most effective CO_2_ absorption, with a tenfold enhancement in adsorption selectivity after LAH treatment. These results highlight how amide functional groups may hinder CO_2_ adsorption by causing pore blockage or forming cyclic rings, but simple modification methods can convert them into effective groups, thereby promoting CO_2_ adsorption.

Amine, a class of organic compounds, consists of molecules that contain one or more nitrogen atoms bonded to hydrogen atoms. Amine-based functionalization has been demonstrated to effectively enhance CO_2_ capture. In a representative example [90], an Al-based MOF named ED@MOF-520 (MOF-520: Al_8_(*μ*_3_OH)_8_(HCOO)_4_(BTB)_4_), integrating ethylenediamine (ED) into its structure, is designed to use for CO_2_ capture. The material demonstrates exceptional performance in CO_2_ capture, with a separation factor of 50 for CO_2_/N_2_ at 273 K, marking a significant increase over the unmodified MOF-520. The presence of amine groups in ED@MOF-520 is shown to enhance interactions with CO_2_ due to the presence of uncoordinated, electron-rich nitrogen atoms within the amine groups. Furthermore, the incorporation of amine groups intensifies the molecular sieving effect by reducing the pore diameter. In addition, the Q_st_ measurements reveal a strong initial interaction between CO_2_ and the amine groups, which is indicative of the material’s affinity for CO_2_. Bose et al. [91] investigated the stability and regeneration conditions of an MOF, specifically N,N-dimethylethylenediamine (mmen) appended Mg_2_(dobpdc). The results show that the mmen-appended Mg_2_(dobpdc) MOF is stable at relative humidities up to 50% when the adsorption temperature ranges from 25–40 °C, with optimal regeneration achievable at 120 °C under dynamic vacuum and at 150 °C under N_2_. The article underscores the potential of this specific MOF material for CO_2_ capture and regeneration in practical applications. In a separate study [92], the incorporation of tetramine groups endows the MOF with a distinctive double-stepped isotherm characteristic, showing great potential for CO_2_ capture from dilute sources. The study delves into the adsorption and desorption behavior of the tetramine-modified MOF under various operating conditions through numerical simulations, considering factors such as isotherm shape, heat transfer coefficient, and feed temperature, which affect the dynamics within a fixed bed. The findings highlight that the tetramine-appended MOF can achieve high purity and recovery rates for CO_2_ capture under isothermal conditions. Despite the advantageous material properties of the tetramine-modified MOF, the steam-assisted temperature swing adsorption (SA-TSA) process results in high steam consumption and low productivity due to long cycle times. This indicates that further optimization of the process design is needed to enhance the overall performance and economic viability when applying this material to practical CO_2_ capture applications. In addition to the above challenges, amine groups can be susceptible to chemical degradation under certain conditions, such as in the presence of oxygen or moisture, which could lead to a reduction in the number of active amine sites over time. Therefore, it is imperative to address this issue in future applications.

In addition to the aforementioned functional groups, the incorporation of -F, -CH_3_, and -Li-O into parent MOFs represents another commonly employed strategy to enhance the materials’ hydrophobicity and interaction with CO_2_ [55,93,94]. 

Fe- and Al-based MOFs are renowned for their controllable pore structure, allowing them to be used in CO_2_ capture and separation. Feng et al. [95] focused on the impact of the pore environment regulated by the SBU on the gas separation performance of MOF membranes for the first time. The Fe_3_(m_3_-O)(CH_3_COO)_6_ SBU of the parent framework (soc-MOF, a stable microporous Fe-MOF) was in situ modified with imidazole (IM) molecules to construct the soc-MOF-IM polycrystalline membrane. The exact location of the incorporated IM and the reduced pore size were observed based on the crystal structure determination. The soc-MOF-IM polycrystalline membrane shows a significantly enhanced H_2_/CO_2_ selectivity, mainly attributed to the triggered molecular sieving effect. Al-based MOFs also show great promise. A microporous Al-based MOF, MIL-120(Al)-AP (AP and P: Institute Lavoisier and Ambient Pressure synthesis, respectively) exhibits high CO_2_ uptake of 1.9 mmol g^−1^ at 0.1 bar and 298 K [96]. In addition, the material’s moderate Q_st_ value of −40 kJ mol^−1^ suggests a low energy penalty for full regeneration. An environmentally friendly ambient pressure synthesis route has been developed for the kilogram-scale production of MIL-120(Al)-AP with high yield, using inexpensive raw materials. The MOF is further shaped into mechanically stable beads with inorganic binders to simulate practical industrial production. This article highlights the potential of MIL-120(Al)-AP in post-combustion CO_2_ capture and provides a comprehensive evaluation of its synthesis, performance, and cost.

### 4.2. MOF Composites

#### 4.2.1. MOF/Ionic Liquids (ILs) Composite

**Figure 12:**
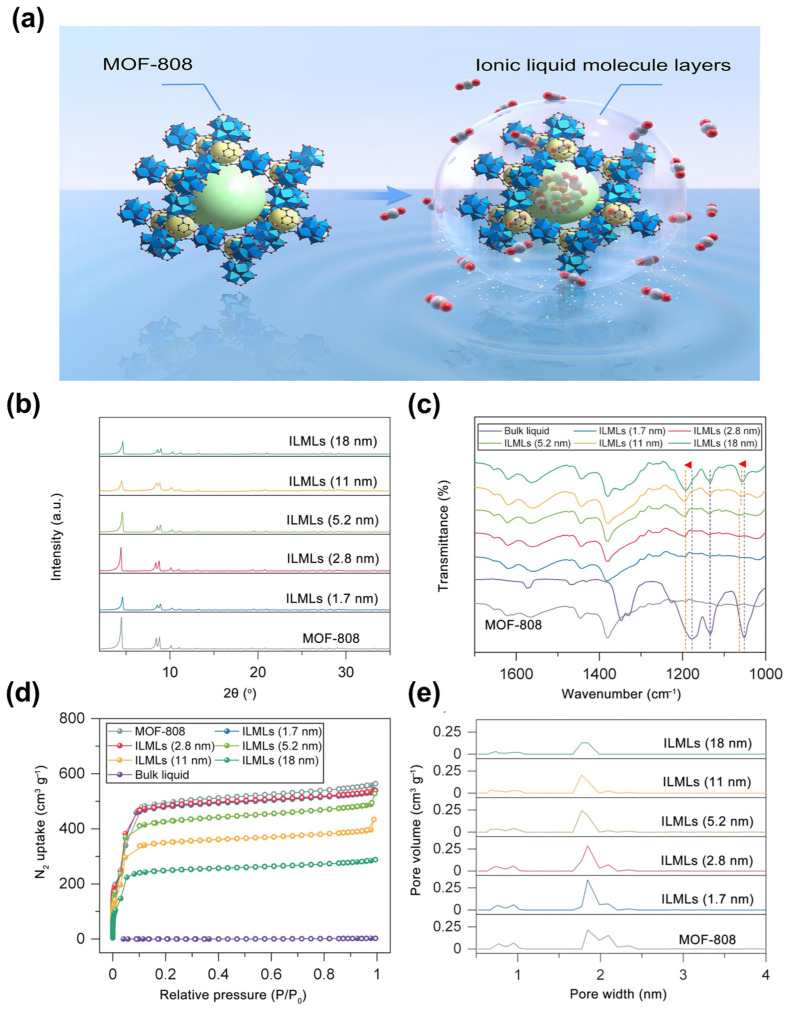
(**a**) Schematic illustration of MOF-808 and MOF/ILMLs. (**b**) PXRD patterns and (**c**) FTIR spectra of pristine MOF-808 and MOF/ILMLs with various thicknesses. (The red arrows represent the blue shift at 1051 cm^−1^ and 1178 cm^−1^, respectively) (**d**) N_2_ adsorption–desorption isotherms of pristine MOF-808 and MOF/ILMLs with various thicknesses. (**e**) Pore size distributions of pristine MOF-808 and MOF/ILMLs with various thicknesses. Reproduced from [97]. Copyright 2022, Elsevier.

ILs are liquids entirely composed of ions. Due to their high thermal stability, low volatility, CO_2_ solubility, and capture capacity, extensive research has been devoted to exploiting the application of ILs. Meng et al. [97] used a solution-mediated assembly method to deposit layers of ionic liquid molecules (ILMLs) onto MOF-808 and evaluated its gas adsorption capability (Figure 12a). MOF-808 crystal powders are treated with methanol and a specific amount of ionic liquid solution to form MOF-808 with ILMLs (MOF/ILMLs). A theoretical thickness of ILMLs ranging from 1.7 nm to 18 nm is observed as a consequence of various IL content additions. The structural integrity after ILML assembly is confirmed by PXRD patterns (Figure 12b). Fourier Transform Infrared Spectrometer (FTIR) analysis (Figure 12c) reveals blue shifts in the symmetric (1051 cm^−1^) and asymmetric stretching vibrations of S=O (1178 cm^−1^), indicating electrostatic interactions between ILML anions and metal nodes, thereby confirming successful assembly of ILMLs on the outer surface of MOF-808. As revealed by N_2_ adsorption–desorption isotherms, MOF/ILMLs exhibit inferior N_2_ uptake compared to MOF-808 (Figure 12d). Although ILMLs are capable of slightly penetrating MOFs’ pore walls and forming an interweaving layer at the IL and MOF-808 interface, the pore structures of MOF/ILMLs are minimally affected (Figure 12e). Due to the shield effect of ILMLs as an adhesive layer and MOF as a gas reservoir, the composites exhibit strong CO_2_ affinity with a nearly zero sorption amount for N_2_. Moreover, Zeeshan et al. [98] designed an IL/UiO-66 (UiO-66: Zr_6_O_6_(OH)_4_(BDC)) composite for infinite CO_2_ selectivity. At lower pressures (100 mbar) and a constant temperature (15 °C), an approximately 455-fold improvement of ideal CO_2_/N_2_ selectivity is observed. Upon the pressure further decreasing to a low-pressure region (1–50 mbar), the ideal selectivity of CO_2_/N_2_ is almost infinite (>100,000).

In summary, ILs have a strong shielding effect on gases such as N_2_ and CH_4_, allowing only CO_2_ to pass through. However, some ILs manifest no CO_2_ adsorption improvement or even reduced CO_2_ uptake because of the existing blockage in the MOF channel. The amount, selection, and incorporation method of ILs should be carefully considered. 

#### 4.2.2. MOF/GO Composite

**Figure 13:**
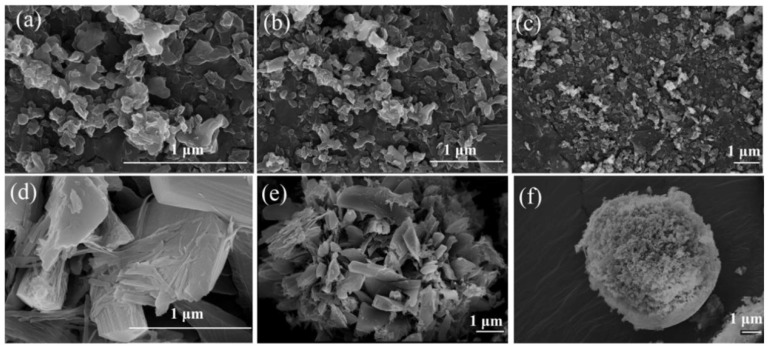
SEM images of (**a**–**c**) Mg/DOBDC prepared by solvothermal method, showing aggregation of flake-like particles. (**d**–**f**) Mg/DOBDC prepared by mechanical stirring method, showing spherical morphology. Reproduced from [99]. Copyright 2021, Elsevier.

**Figure 14:**
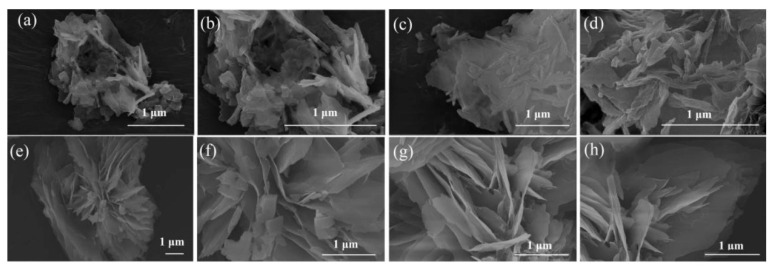
SEM images of (**a**–**d**) Mg/DOBDC MOF@GO_W-30_ composite, showing a bird’s nest morphology. (**e**–**h**) Mg/DOBDC MOF@GO_D-40_ composite, showing a lotus morphology. Reproduced from [99]. Copyright 2021, Elsevier.

GO is a graphitic material that can be integrated with MOF to enhance CO_2_ adsorption performance. Wang et al. [99] provided a comprehensive investigation into the synthesis and application of MOF@GO composites, focusing on Mg/DOBDC MOF (consisting of Mg(II) ions linked by 2,5-dioxido-1,4-benzenedicarboxylate (DOBDC) ligands) coupled with GO. The study examined the influence of various factors, including the preparation method of the MOF, the solvent used for synthesizing GO, and the volume ratio of GO in the composite. Specifically, two distinct methods were employed for synthesizing Mg/DOBDC MOF: solvothermal and mechanical stirring. Moreover, GO was synthesized and delaminated in either deionized water (GO_w_) or N, N-Dimethylformamide (DMF) (GO_D_) solvent. Different volumes of GO (20 mL, 30 mL, 40 mL, and 50 mL, denoted as GO_20_, GO_30_, GO_40_, and GO_50_) were added into the mixture and reacted with Mg/DOBDC to obtain the final Mg/DOBDC MOF@GO composite. As a result, scanning electron microscopy (SEM) demonstrates significant differences in morphology between Mg/DOBDC samples prepared by different methods, as well as between MOF@GO composites synthesized using different GO solvents and volumes (Figure 13 and Figure 14). Further CO_2_ adsorption performance tests confirm that the adsorption capacity of Mg/DOBDC MOFs prepared by different methods is almost non-significant. The CO_2_ adsorption and penetration are strongly related to the GO volumes and followed the order of Mg/DOBDC MOF@GO_W-40_ < Mg/DOBDC MOF@GO_W-50_ < Mg/DOBDC MOF@GO_W-20_ < Mg/DOBDC MOF@GO_W-30_. The optimal composite, Mg/DOBDC MOF@GO_W-30_, exhibits nearly twice the CO_2_ uptake of the parent MOF, reaching 8.60 mmol g^−1^ at 0.1 MPa and 25 °C. In contrast, Mg/DOBDC MOF@GO_D_ with the optimal GO volume shows lower CO_2_ uptake under the same conditions, indicating better improvement when using water as the solvent for GO synthesis. The mechanism underlying the enhanced performance of the MOF@GO composites involves the development of a pore structure conducive to CO_2_ adsorption after GO incorporation and the exposure of additional active sites through coordination between functional groups of GO and metal sites within the MOF. 

#### 4.2.3. Other MOF Composites

**Figure 15:**
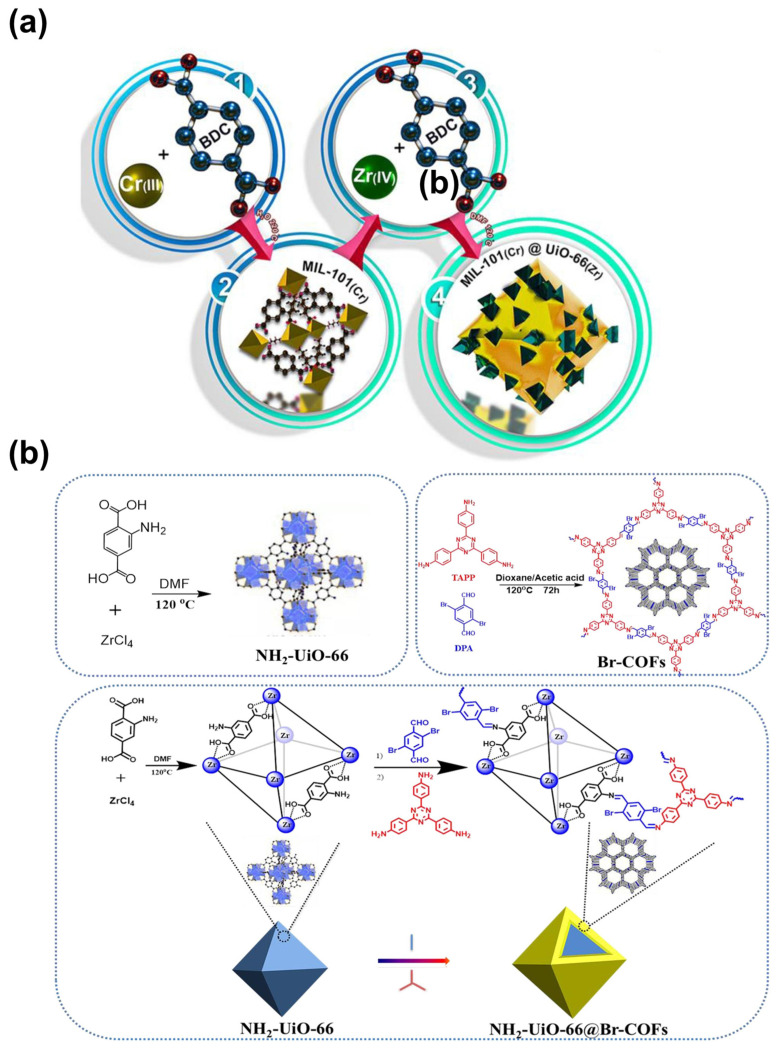
Synthetic procedure of (**a**) MU. Reproduced with permission from [100]. Copyright 2023, Elsevier. (**b**) NH_2_-UiO-66@Br-COFs. TAPP: 4,4′,4″-(1,3,5-triazine-2,4,6-triyl) trianiline, DPA: 2,5-dibromo-1,4-diformylbenzene. Reproduced with permission from [102]. Copyright 2022, Elsevier.

Although many MOFs exhibit exceptional CO_2_ adsorption capacities, they often face stability issues. Conversely, MOFs that demonstrate good thermal or chemical robustness frequently lack substantial CO_2_ adsorption capabilities. To address these challenges, many efforts have been dedicated to combining two MOFs together to improve their properties. A notable example is the core–shell structure of MIL-101(Cr)@UiO-66(Zr) (denoted as MU), assembled from Cr_3_F(H_2_O)_2_O(BDC)_3_·nH_2_O (MIL-101(Cr)) and UiO-66(Zr) crystals (Figure 15a) [100]. This combination leads to improved thermal stability and increased OMSs. Most importantly, an optimal pore diameter is achieved, resulting in minimal uptake of CH_4_ and N_2_ but enhanced uptake for CO_2_, which contributes to the superior selectivity of CO_2_/CH_4_ and CO_2_/N_2_.

Additionally, integrating MOFs with biochar significantly enhances CO_2_ adsorption capacity. For instance, grafting Zn(MeIm)_2_ (ZIF-8, MeIm: methylimidazole) onto a biochar to form nitrogen-enriched modified biochar has shown promise [101]. Through in situ growth and annealing at temperatures ranging from 300 °C to 500 °C, the resultant MOF/biochar composite shows an obvious improvement in surface area and pore volume. Optimal CO_2_ adsorption capacity, regenerability, and stability are achieved with a biochar quantity of 0.5 g and annealing at 400 °C, attributed to increased microporosity and pyrrolic nitrogen content. 

Other composites such as MOF@COF and MOF@porous carbon have also demonstrated enhanced properties. For example, a core–shell like NH_2_-UiO-66@Br-COFs performs better compared to single MOF and COF, thanks to the newly produced ultramicropores at the interface [102]. The synthetic procedure is presented in Figure 15b. Liu et al. [103] developed three novel composites by merging an MOF framework and porous carbon. All the as-prepared materials inherit the advantageous structure properties from both parent materials and produce noticeably higher specific surface area and additional micropores.

**Table 2:** Pristine MOFs for CO_2_ capture and separation and their performance.

MOF	Adsorbed Amount (mmol g^−1^)	Pressure (kPa)	Gas Separation	IAST Selectivity (298 K, 100 kPa)	Ref.
([Co_5_(μ_3_-OH)_2_(aip)_4_(H_2_O)_2_])(MUF-17)	2.5	100	CO_2_/N_2_	501	[83]
[Cu_2_(BPDC)_2_(H_2_O)_2_]·5.6H_2_O·DMA	8.6	100	CO_2_/N_2_	41.5	[104]
[(CH_3_)_2_NH_2_][Zn_1.5_(μ_3_-O)_0.5_(F-tzba)_1.25_(bpy)_0.25_(μ_2_-F)_0.5_]·2DMF·2H_2_O	2.1	100	CO_2_/CH_4_	4.9	[105]
{[PbZn(L)_2_]·DMA·H_2_O}_n_ (HMOF)	1.8	100	CO_2_/CH_4_	16.3	[86]
In(aip)_2_	1.3	101	CO_2_/N_2_	/	[59]
Cu-OATA	6.2	100	CO_2_/N_2_	/	[51]
MOF-808-EDTA-ED-R	1.6	100	CO_2_/N_2_	/	[89]
MOF-177-DETA	2.8	100	/	/	[52]
MOF-177-PEI	2.8	100	/	/	[52]
MOF-177-TEPA	4.6	100	/	/	[52]
ZnMOF	1.7	100	CO_2_/CH_4_	6.16	[106]
[Zr_6_O_4_(OH)_6_(H_2_O)_2_ (TBAPy)_2_(PhTz)]Br (NU-1000-PhTz)	1.4	100	CO_2_/N_2_O	1.1	[107]
F-Ni-MOF-74	/	1500	CO_2_/CH_4_	/	[55]
Li-O-Ni-MOF-74	195.6	1500	CO_2_/CH_4_	/	[55]
NH_2_-Ni-MOF-74	183.6	1500	CO_2_/CH_4_	/	[55]
CH_3_-Ni-MOF-74	160.3	1500	CO_2_/CH_4_	/	[55]
MOF-808-glycine	0.5	15	/	/	[87]
MOF-808-sarcosine	0.6	15	/	/	[87]
MOF-808-L-alanine	0.5	15	/	/	[87]
MOF-808-DL-alanine	0.5	15	/	/	[87]
MOF-808-(R)-3-aminobutanoic acid	0.3	15	/	/	[87]
MOF-808-(RS)-3-aminobutanoic acid	0.4	15	/	/	[87]
MOF-808-L-isoleucine	0.4	15	/	/	[87]
MOF-808-L-serine	0.6	15	/	/	[87]
MOF-808-L-threonine	0.3	15	/	/	[87]
MOF-808-L-histidine	0.3	15	/	/	[87]
MOF-808-DL-lysine	1	15	/	/	[87]
M-808-TEPA(0.3)	1.1	100	CO_2_/N_2_	84.4	[108]
M-808-TEPA(0.5)	1.2	100	CO_2_/N_2_	92.9	[108]
M-808-TEPA(1.0)	1.1	100	CO_2_/N_2_	174	[108]
M-808-TEPA(2.0)	1.3	100	CO_2_/N_2_	256	[108]
M-808-TEPA(2.5)	1	100	CO_2_/N_2_	156	[108]
M-808-ED(0.3)	1.2	100	CO_2_/N_2_	48.4	[108]
M-808-DETA(0.3)	0.7	100	CO_2_/N_2_	52.7	[108]

**Table 3:** MOF composites for CO_2_ capture and separation and their performance.

MOF	Adsorbed Amount (mmol g^−1^)	Pressure (kPa)	Gas Separation	IAST Selectivity (298 K, 100 kPa)	Ref.
CuBTC@1%GO	8.9	100	CO_2_/N_2_	/	[35]
HKUST-1@10UV-GO	5.14	100	CO_2_/N_2_	2.53	[109]
Mg/DOBDC MOF@GOw-30	8.6	100	/	/	[99]
MOF-808 with ILMLs	3	100	CO_2_/N_2_	478	[97]
UiO-67-ILs-Cl	3.8	101	CO_2_/N_2_	44.21	[110]
NH_2_-UiO-66@Br-COF-1	1.9	100	CO_2_/N_2_	/	[102]
NH_2_-UiO-66@Br-COF-2	2	100	CO_2_/N_2_	27	[102]
NH_2_-UiO-66@Br-COF-3	2.8	100	CO_2_/N_2_	24.92	[102]
NH_2_-UiO-66@Br-COF-4	3.9	100	CO_2_/N_2_	24.08	[102]
MIL-101(Cr)@UiO-66(Zr) (MU-4)	2.4	100	CO_2_/CH_4_	7.64	[100]
MIL-101(Cr)@UiO-66(Zr) (MU-8)	2.4	100	CO_2_/CH_4_	7.74	[100]

## 5. MOF-Based Materials for CO_2_ Photocatalytic Reduction

Semiconductors (SC) are recognized for their capacity to produce photo-induced carriers upon exposure to sunlight. When photons possess energy equal to or greater than the band gap of the SC, electrons in the VB can absorb these photons and are thereby excited to the CB, leading to the formation of electron–hole pairs. However, traditional SC catalysts face limitations such as large band gaps, restricted visible light adsorption, low charge separation efficiency, and lack of definite structure [111], hindering their practical application.

In alignment with the behavior of SC, MOF has attracted a lot of attention as an alternative for SC. On one hand, as discussed in the adsorption section, MOF itself has a unique porous structure and high surface area, which is conducive to CO_2_ adsorption. On the other hand, the well-defined metal nodes/clusters and organic linkers within MOFs can serve as active sites and light antenna units, respectively [112]. The strong CO_2_ adsorption capacity of MOFs allows CO_2_ to access the active sites, and the homogeneous distribution of metal centers promotes the CO_2_ reduction process. 

The performances of various MOF-based materials, including pristine MOFs and MOF composites are illustrated in Table 4 and Table 5, respectively.

### 5.1. Pristine MOFs

Porphyrin, the core component of chlorophyll, possesses the remarkable ability to sense photons and transmit electrons. Inspired by the uniqueness of porphyrin, integrating porphyrin ligands into MOFs to enhance light-harvesting capacity and promote efficient separation of electron–hole pairs is reckoned as a viable strategy. Numerous porphyrin-based MOFs have already achieved great performance in CO_2_ photocatalytic reduction.

**Figure 16:**
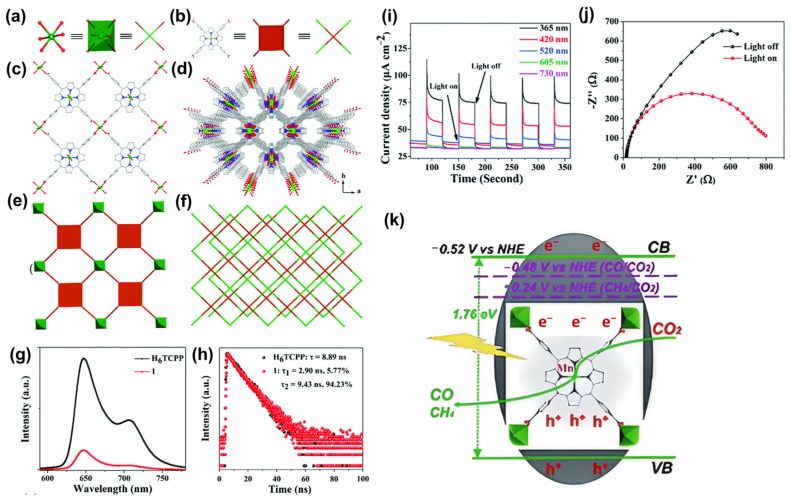
Crystal structure and underlying network topology of Mn-MOF. (**a**) The Mn ions (green octahedra) are linked by four Mn-TCPP ligands ((**b**): brown squares) to form a 2D (4,4)-connected topology, shown in (**c**,**e**). A top view of the stacked 2D layers is shown in (**d**,**f**). Mn atoms are represented by green spheres, C by gray, O by red, and N by blue. H atoms have been omitted for clarity. (**g**) PL emission excitation at 520 nm and (**h**) time-resolved PL kinetics at 706 nm. (**i**) Transient photocurrent responses under varied monochromatic light. (**j**) EIS of **1** measured at a bias potential of 1.4 V vs. Ag/AgCl. (**k**) The reaction mechanism of **1**. Reproduced from [113]. Copyright 2021, Royal Society of Chemistry.

Recently, a novel two-dimensional porphyrin-based Mn-MOF [BMI]_2_{Mn[Mn(H_2_O)_2_-TCPP](H_2_O)_2_} (**1**) based on *meso*-tetra(carboxyphenyl) porphyrin (H_6_TCPP) with atomically dispersed unsaturated porphyrin Mn sites was proven to demonstrate superior activity in CO_2_ reduction [113]. An ionic liquid [BMI]Br was used in the synthetic procedure to ensure high loading of Mn(II)-metalated porphyrin (Mn-TCPP) sites. Each Mn-TCPP ligand is connected with four mononuclear Mn(II) ions, extending in the *bc* plane to form 2D layers in a non-interpenetrated AB stacking pattern (Figure 16a–f). The pores’ dimensions are estimated to be about 7.49 × 13.81 Å^2^ along the *c* axis and 45.2% void space is occupied by [BMI]^+^ cations. Photocatalytic experiments were carried out in a top-irradiation vessel with water vapor. Under 4 h of light illumination, CH_4_ is detected as the main product, with a production rate of 53 μmol h^−1^ g^−1^, while a limited amount of CO is also produced with a production rate of 21 μmol h^−1^ g^−1^. To evaluate the optical properties of the materials, steady-state photoluminescent (PL) measurements, incident-photon-to-current conversion efficiency (IPCE) characterization, and electrochemical impedance spectroscopy (EIS) measurements were conducted for the H_6_TCPP and **1**. Correspondingly, H_6_TCPP exhibits significant quenching of PL intensity (Figure 16g), and **1** exhibits short-lived *τ*_1_ (Figure 16h), high photocurrent response (Figure 16i), and small impedance value (Figure 16j), indicating efficient charge separation ability. The mechanism involves Mn-TCPP acting as a light-harvesting unit for generating electron–hole pairs, and then the photo-induced electrons transfer to the catalytic Mn(II) centers for further CO_2_ reduction (Figure 16k). This work sheds light on the possibility of fabricating porphyrin-based MOFs for CO_2_ photoreduction in gas–solid conditions without using organic sacrificial agents.

**Figure 17:**
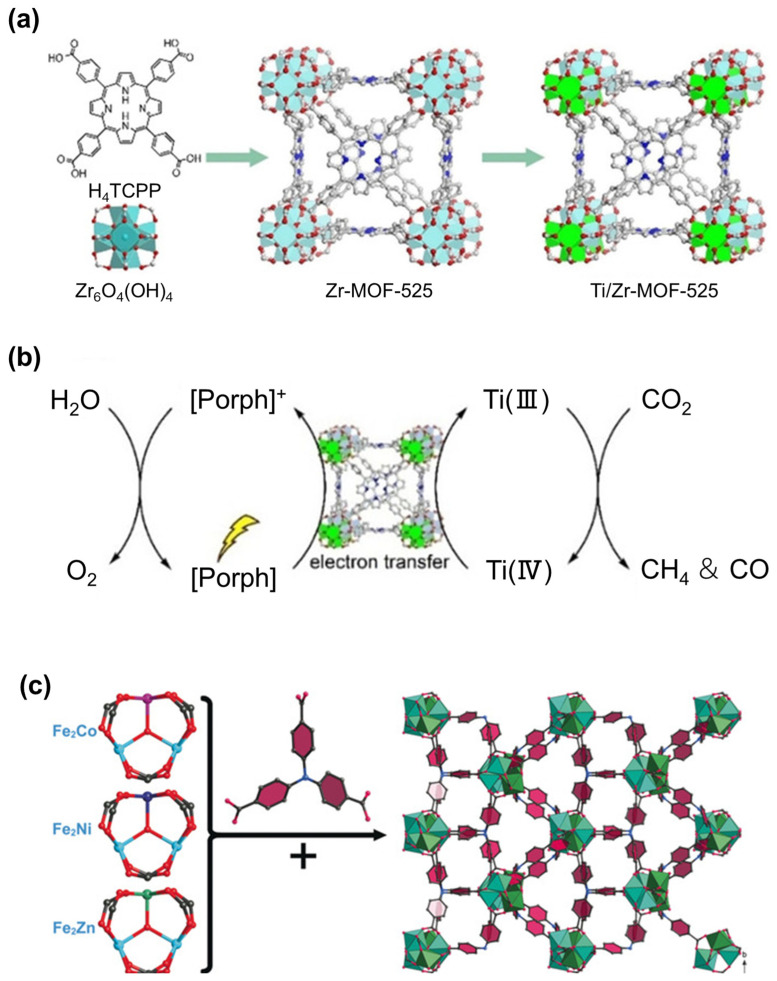
(**a**) Synthetic procedure of Ti/Zr-MOF-525 (obtained by metal metathesis with Ti in the precursor Zr-MOF-525). (**b**) Electron transfer pathway and CO_2_ photoreduction processes of Ti/Zr-MOF-525. Reproduced from [114]. Copyright 2021, Wiley. (**c**) Schematic illustration of NNU-31-M constructed by Fe_2_M cluster and TCA ligand. Reproduced from [115]. Copyright 2020, Wiley.

Despite the prevalent use of monometallic centers, the current trend indicates a shift toward designing bimetallic or mixed-metal centers. In 2020, Gao et al. [114] reported a mixed-metal porphyrinic framework Ti/Zr-MOF-525, synthesized by partially exchanging Zr with Ti in Zr-MOF-525 (Zr-MOF-525 consisted of Zr_6_O_4_(OH)_4_ units and H_4_TCPP ligand) for CO_2_ photoreduction to CH_4_ (Figure 17a). The partial replacement of Ti boosts the electron transport capacity of the entire system. The electron transfer is facilitated to the Ti-containing units for the production of CH_4_ (Figure 17b). Similarly, Dong et al. [115] reported the synthesis of heterometallic Fe_2_M cluster-based MOFs ([Fe_2_M(μ_3_-O)(TCA)_2_(H_2_O)_3_] (M = Co, Ni, Zn), NNU-31-M) for achieving artificial photosynthetic overall reaction without the need for additional sacrificial agents and PS. These MOFs are constructed by Fe_2_M(μ_3_-O)(OAc)_6_(H_2_O)_3_ clusters and photosensitive TCA ligands (Figure 17c). Among all the heterometallic cluster-based MOFs, NNU-31-Zn exhibits the best performance, with HCOOH yield of 26.3 μmol h^−1^ g^−1^ and selectivity of 100%. The matched band structures, weaker PL signals, and stronger photocurrent response account for the fast electron–hole separation. Further DFT calculations reveal that Zn and Fe sites play distinct roles in the reaction. Zn sites favor CO_2_ conversion into HCOOH due to a relatively low energy barrier to the rate-determining step (*CO_2_ to *OCHO), while Fe sites are responsible for water oxidation to oxygen. The positive synergistic effect of Zn and Fe is the main reason for the performance improvement. Obviously, the above examples offer a modification method through building two different metal sites. Furthermore, modification through developing the mixed-valence state of the metal center is another feasible way, especially for Cu-based MOFs. The mixture of Cu(I)/Cu(II) in MOF can positively affect the binding energy of the intermediates [116].

### 5.2. MOF Composites

The incorporation of catalytically active entities, including metal NPs/single-atom (SAs)/nanoclusters (NCs), PS, SCs, or quantum dots (QDs), into MOFs to fabricate MOF composites represents a widely endorsed strategy for the photocatalytic reduction of CO_2_ [117]. The integrated species not only broaden the visible light response range but also serve as active sites for CO_2_ activation. Additionally, they function as platforms for electron migration, thereby enhancing charge transfer efficiency and CO_2_ production rate.

#### 5.2.1. MOF/Encapsulated Metal Composite

**Figure 18:**
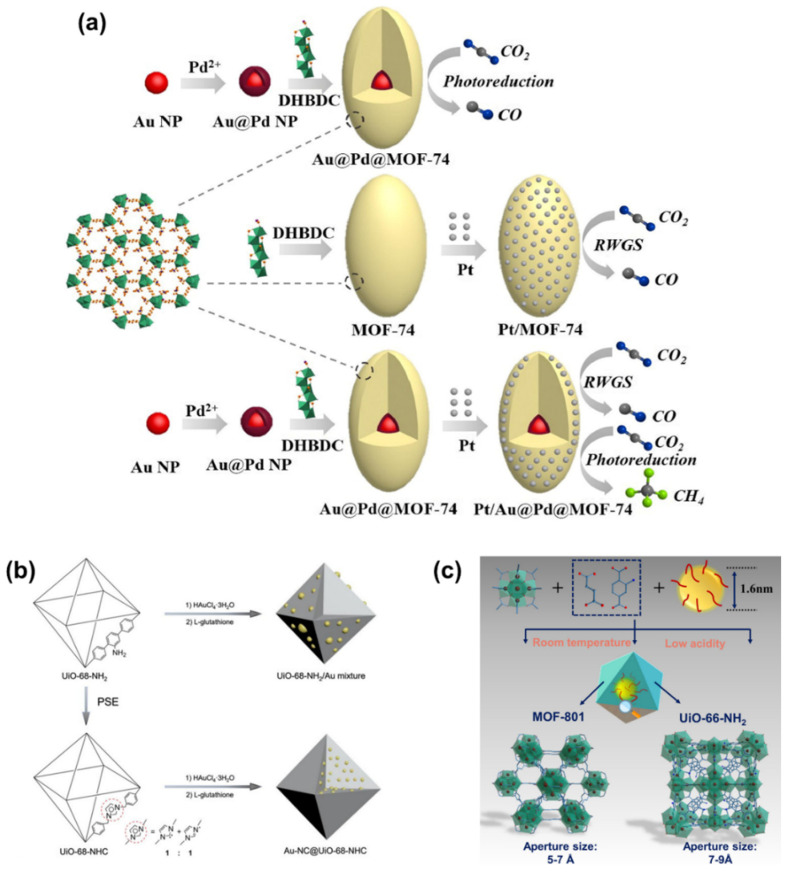
(**a**) The assembly of Au@Pd@MOF-74, Pt/MOF-74, Pt/Au@Pd@MOF-74. Au@Pd@MOF-74 for photoreduction of CO_2_, Pt/MOF-74 only for RWGS of CO_2_ and Pt/Au@Pd@MOF-74 both for RWGS and photoreduction of CO_2_. DHBDC: 2,5-dihydroxybenzene-1,4-dicarboxylic acid. Reproduced from [119]. Copyright 2019, Elsevier. Schematic illustration of the synthetic process of (**b**) UiO-68-NHC, UiO-68-NH_2_/Au mixture, and Au NCs incorporated into UiO-68-NHC. Reproduced from [121]. Copyright 2021, Wiley. (**c**) Cu NCs incorporated into MOF-801 and UiO-66-NH_2_ and their aperture sizes. Reproduced from [120]. Copyright 2022, Wiley.

The integration of encapsulated metals, including metal NPs, NCs, and SAs with MOF, offers a platform to leverage the distinctive attributes of each constituent, fostering a collective and synergistic effect [118]. Beyond the interaction between MOF and encapsulated metals, noteworthy synergies also emerge among multiple integrated metals. An illustrative example is found in Au@Pd@MOF-74 [119], where core–shell Au@Pd NPs are embedded within MOF-74, and Pt NPs are further coated onto the surface of MOF-74, resulting in Pt/Au@Pd@MOF-74. In comparison, Pt/MOF-74 is constructed comprising Pt NPs solely loaded onto the surface (Figure 18a). The CH_4_ yield is contrasted on these two materials. As a result, the CH_4_ yield over Pt/Au@Pd@MOF-74 reaches 12.35 μmol g^−1^, with a CH_4_ selectivity of 84%, outperforming that of monometallic NP-doped Pt/MOF-74. Each component plays a crucial role in the catalyst’s efficacy. MOF-74 serves as an electron adsorption and transport medium, Au@Pd NPs help to improve the photon adsorption capacity, and Pt NPs are capable of capturing more electrons. Absence of Pt NPs results in the sole production of CO instead of the desired CH_4_, while the catalyst’s photocatalytic activity is compromised in the absence of Au@Pd NPs’ encapsulation.

Furthermore, significant progress has been made in integrating NCs and SAs into MOFs [117,120,121]. For instance, ultrasmall and highly dispersed Au NCs are stabilized within Zr_6_O_6_(OH)_4_(TPDC) (UiO-68, TPDC: terphenyl-4,4″-dicarboxylate) using N-heterocyclic carbene (NHC) ligands via a heterogeneous nucleation approach (Figure 18b) [121]. Identically, Cu NCs are steadily incorporated into UiO-66-NH_2_ and Zr_6_O_4_(OH)_4_(fumarate)_6_ (MOF-801) via a scalable room temperature fabrication approach (Figure 18c) [120]. Upon successful incorporation, both materials exhibit remarkable efficacy in CO_2_ photocatalytic reduction. Moreover, Hao et al. [122] also conducted a comparative study on the catalytic performance of NPs, NCs, and SAs within an MOF membrane. The photocatalytic CO_2_ reduction efficiency to HCOOH follows the order of: Ir_NPs_ < Ir_NCs and SAs_/ < Ir_SAs_. This divergence can be attributed to the differences in their electronic structures. Unlike Ir NPs and Ir clusters, Ir SAs exhibit a more positive charge, resulting in a relatively lower apparent activation energy for HCOOH evolution. This feature favors HCOOH formation and accelerates the reaction kinetics.

#### 5.2.2. MOF/PS Composite

In photocatalysis, the integration of a PS agent is crucial to facilitate the desired reaction. Upon absorption of photons, the PS molecule transitions to an excited state, denoted as PS*. This excited state subsequently undergoes reduction, forming the PS^−^ species as it gains electrons from a sacrificial donor. The PS^−^ then donates these electrons to the catalytic center, thereby participating in the photocatalytic process, and subsequently reverts to its initial ground state. The Ru-polypyridyl complex is one of the most commonly used PS agents. Co-integrating Ru and Re as a PS and active catalyst has been validated as a useful strategy for promoting electron transfer between the PS and active catalyst [123,124]. However, due to their high cost and toxicity, the use of Ru-polypyridyl complexes is considered environmentally unfriendly. Therefore, the search for a cheap and earth-abundant element to replace Ru is of great importance. Given that Cu is easily available and cost-effective, it is regarded as one of the ideal alternatives. 

**Figure 19:**
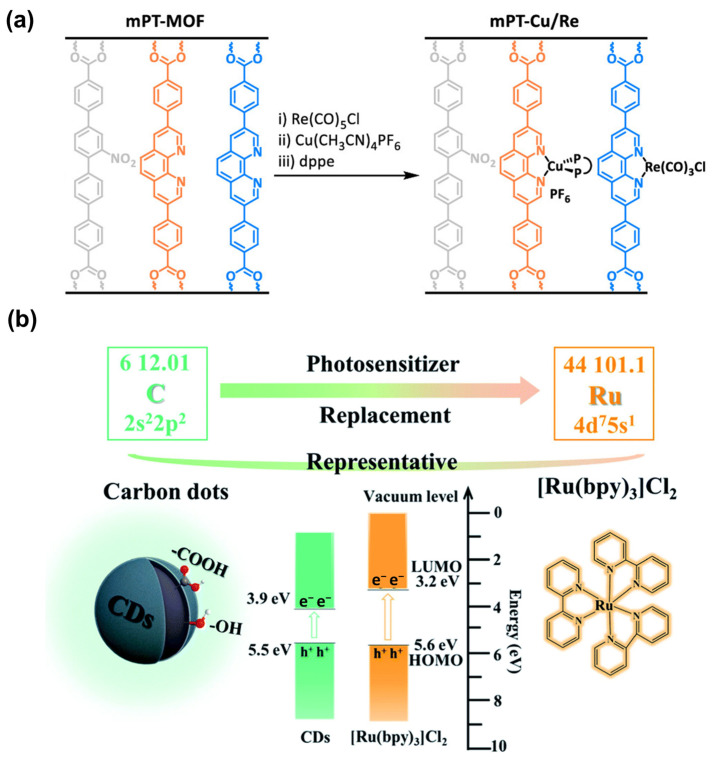
(**a**) Schematic illustration of the synthetic process of mPT-Cu/Re. Reproduced from [125]. Copyright 2020, American Chemical Society. (**b**) Comparison between noble metal Ru complex and CDs in terms of resource consumption and economic cost. Reproduced from [36]. Copyright 2022, Royal Society of Chemistry.

In pursuit of this goal, mPT-Cu/Re was synthesized based on mPT-MOF with mixed phenanthroline dibenzoate (PT) and 2′′-nitro-[1,1′:4′,1′′:4′′,1′′′-quaterphenyl]-4,4′′′-dicarboxylate (TPHN) ligands through stepwise metalation with Cu(CH_3_CN)_4_PF_6_ and Re(CO)_5_Cl (Figure 19a) [125]. In mPT-Cu/Re, Cu^I^ centers coordinate with PT and 1,2-bis(diphenylphosphino)ethane (dppe) in a distorted tetrahedral geometry, while Re centers form an octahedral geometry coordinating with PT, three carbonyl groups, and a chlorine atom. Photocatalytic experiments were carried out under 350–700 nm light irradiation with BIH as sacrificial agent. With the assistance of Cu-PS, mPT-Cu/Re exhibits a high turnover number (TON) and CO/H_2_ selectivity, indicating the high solar energy conversion ability of Cu-PS. 

Moreover, carbon dots (CDs) emerge as a viable alternative, distinguished by their cost-effectiveness and capacity for electron storage and transfer. The merits of CDs can be divided into three facets: (1) broadened visible light adsorption range, (2) increased exposure of active sites, and (3) enhanced charge transfer efficiency. Qian et al. [36] compared the CO_2_ photocatalytic activity of a CD-based catalyst with a Ru-complex-based catalyst (Figure 19b). Surprisingly, under the same conditions, the CD-based catalyst exhibits a better CO generation rate than the Ru-complex-based catalyst, outperforming the Ru-complex-based catalyst from both economic and efficiency perspectives. Consequently, replacing the Ru complex with CDs represents a promising and economical way to fabricate PS-containing MOF materials.

#### 5.2.3. MOF/SC Composite

**Figure 20:**
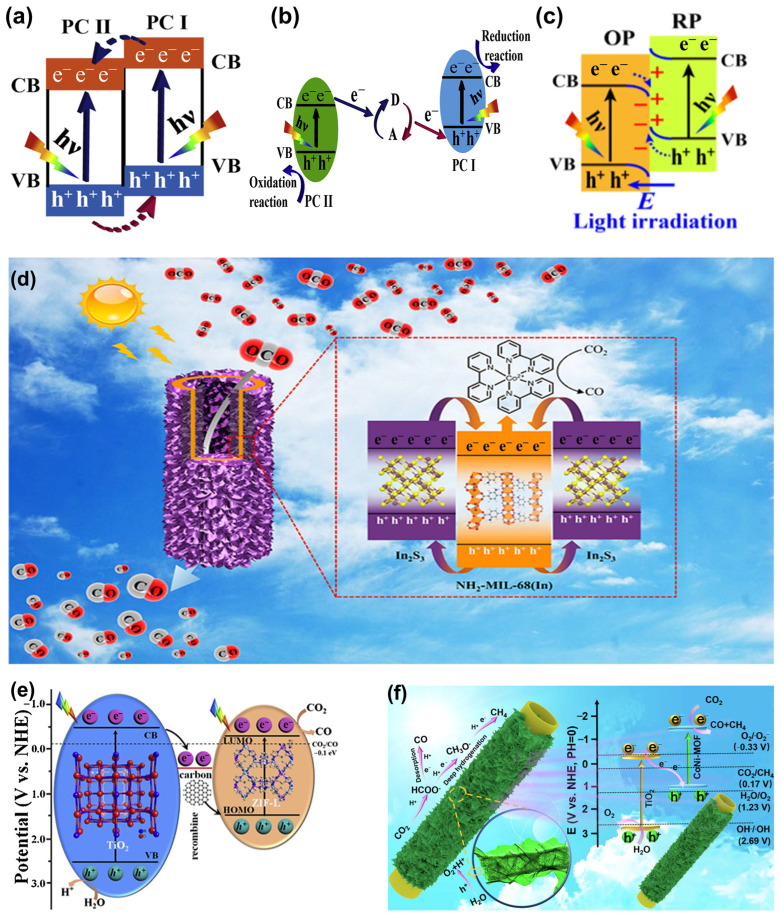
Schematic illustration of charge transfer in heterojunctions. (**a**) Charge transfer pathway of type-II heterojunction. (**b**) Charge transfer pathway of Z-scheme heterojunction. (**c**) Charge transfer pathway of S-scheme heterojunction. Reproduced from [129]. Copyright 2020, Elsevier. Schematic illustration of the mechanism of (**d**) type-II catalyst In_2_S_3_@NH_2_-MIL-68(In)@In_2_S_3_. Reproduced from [126]. Copyright 2020, American Chemical Society. (**e**) Z-scheme catalyst TiO_2_/C/MOF. Reproduced from [127]. Copyright 2020, Elsevier. (**f**) S-scheme catalyst TiO_2_@CoNi-MOF. Reproduced from [128]. Copyright 2023, Elsevier.

The strategic assembly of MOFs with SCs to engineer heterojunctions presents an effective approach to accomplish the separation of electron–hole pairs. According to the mechanism of electron transfer pathways, heterojunctions engineered by integrating MOF with SC can be classified into three categories. The first category is type-II heterojunctions. As shown in Figure 20a, type-II heterojunctions generally exhibit higher VB and CB energies in photocatalyst (PC) I than in PC II. Due to the disparity in band energy potential between the two PCs, an internal electric field is built, which drives the migration of photo-induced electrons and holes. Photo-induced electrons in the CB of PC I transfer to the CB of PC II, while photo-induced holes in the VB of PC II migrate to the VB of PC I. Hence, effective separation of electron–hole pairs is achieved. In a typical example [126], the electron–hole transfer pathway on a sandwich homologous heterojunction constructed by In_2_S_3_@NH_2_-MIL-68(In)@In_2_S_3_ (denoted as SMS(In)) follows type-II mechanism. Electrons from CB of In_2_S_3_ migrate and accumulate in the CB of NH_2_-MIL-68(In), while holes from VB of NH_2_-MIL-68(In) transfer to VB of In_2_S_3_ (Figure 20d). Benefiting from the heterostructure, SMS(In) demonstrates a high CO production rate under visible light irradiation. However, traditional type-II heterojunctions suffer from certain limitations. On one hand, the charge transfer pathway is thermodynamically unfavorable. On the other hand, the CB and VB with strongest redox abilities cannot be retained. 

In this regard, Z-scheme heterojunctions have been proposed, using a mediator to capture the separated electrons and holes from the lower redox potential of each SC. This ensures that the most potent redox electrons and holes are retained (Figure 20b). A ternary composite TiO_2_/C/MOF [127], consisting of carbonized TiO_2_/PAN (PAN: polyacrylonitrile) fibers and a leaf-like zeolitic imidazolate frameworks (ZIF-L), represents a typical Z-scheme heterojunction. The photo-induced electrons from CB of TiO_2_ first migrate to the carbon and then recombine with the holes from VB of ZIF-L, hence enabling the electrons to be retained in the lowest unoccupied molecular orbital of ZIF-L (Figure 20e). Although Z-scheme heterojunctions preserve the reduction ability of electrons, the issue of unfavorable thermodynamic transfer pathways remains unaddressed. 

Currently, the concept of constructing S-scheme heterojunctions is proposed to overcome such a drawback. S-scheme heterojunctions are composed of an oxidation PC and a reduction PC, each featuring staggered band structures that facilitate charge separation. Upon contact, the band bending occurs as a result of the Fermi level disparity between the two PCs, establishing an internal electric field. This field persists until the Fermi levels equilibrate. The Coulombic forces generated by this electric field drive the recombination of non-essential photo-induced charge carriers, while simultaneously ensuring the effective segregation of potent electrons and holes (Figure 20c). Liang et al. [128] reported a TiO_2_@CoNi-MOF hierarchical hollow nanotube for efficient CO_2_ photoreduction to methane. An S-scheme mechanism is proposed, where the synergy of Coulomb interaction, internal electric field and interface band bending facilitates the corresponding charge carriers’ recombination and separation (Figure 20f). To sum up, S-scheme heterojunctions not only incorporate the advantages of type-II and Z-scheme heterojunctions but also offer a more rational electron transfer route.

**Table 4:** Pristine MOFs for CO_2_ photocatalytic reduction and their performance.

MOF	Metal Node/Cluster	Main Product	Photocatalytic Efficiency (μmol h^−1^ g^−1^)	Selectivity (%)	Ref.
[BMI]_2_{Mn[Mn(H_2_O)_2_-TCPP](H_2_O)_2_}cc	Mn^2+^	CH_4_	53	71.62	[113]
NNU-55-Ni	Ni^2+^	CO	266.6	81	[130]
NNU-31-Zn	Fe_2_Zn cluster	HCOOH	26.3	100	[115]
NNU-31-Co	Fe_2_Co cluster	HCOOH	14.94	/	[115]
NNU-31-Ni	Fe_2_Ni cluster	HCOOH	17.13	/	[115]
PCN-250-Fe_3_	Fe_3_ cluster	CO	13,450	/	[131]
PCN-250-Fe_2_Mn	Fe_2_Mn cluster	CO	21,510	/	[131]
PCN-250-Fe_2_Zn	Fe_2_Zn cluster	CO	19,450	/	[131]
PCN-250-Fe_2_Ni	Fe_2_Ni cluster	CO	15,860	/	[131]
PCN-250-Fe_2_Co	Fe_2_Co cluster	CO	14,010	/	[131]
Ti/Zr-MOF-525	Ti/Zr	CH_4_	1.52	/	[114]

**Table 5:** MOF composites for CO_2_ photocatalytic reduction and their performance.

MOF	Integrated Species	Main Product	Photocatalytic Efficiency (μmol h^−1^ g^−1^)	Selectivity (%)	Ref
MOF/NPs/SAs/NCs					
IrNPs/A-aUiO	Ir NPs	HCOOH	10	16.5	[122]
Ir_x_/A-aUiO	Ir NCs/Ir SAs	HCOOH	160	79.8	[122]
Ir_1_/A-aUiO	Ir SAs	HCOOH	240	96.3	[122]
Au@Pd@MOF-74	Au@Pd NPs	CO	2.46	100	[119]
Pt/Au@Pd@MOF-74	Au@Pd NPs, Pt NPs	CH_4_	2.47	84	[119]
MIL-101(Cr)/Ag NPs (80MA)	Ag NPs	CO	808.2	/	[132]
MIL-101(Cr)/Ag NPs (150MA)	Ag NPs	CO	298.5	/	[132]
MIL-101(Cr)/Ag NPs (400MA)	Ag NPs	CO	49.1	/	[132]
MIL-101(Cr)/Ag NPs (800MA)	Ag NPs	CO	34	/	[132]
Au-NC@UiO-68-NHC	Au NCs	CO	57.6	/	[121]
Cu NCs@UiO-66-NH_2_	Cu NCs	HCOOH	128	86	[117]
Cu SAs/UiO-66-NH_2_	Cu SAs	CH_3_OH	5.33	/	[117]
MOF/PS					
(Co/Ru)_2.4_-UiO-67(bpydc)	Co/Ru	syngas	850	/	[133]
Zr-MBA-Ru/Re-MOF	Ru/Re	CO	440	>99	[123]
Ni3@Ru-UiO-67	Ni^2+^ complex/Ru	CO	426.05	>99	[134]
mPT-Cu/Re	Cu/Re	CO	11,000	89	[125]
MOF/SC					
CsPbBr_3_ QDs/UiO-66(NH_2_)	CsPbBr_3_	CO	8.21	/	[135]
CTU(CuTCPP)/0.6TiO_2_	CuTCPP/TiO_2_	CO	31.32	/	[136]
TiO_2_/C@ZnCo-ZIF-L	TiO_2_/C	CO	28.6	99	[127]
UiO-66-NH_2_/Cu_2_O/Cu-0.39	Cu_2_O/Cu	CO	4.54	/	[137]
Ce-MOF/Bi_2_MoO_6_	Bi_2_MoO_6_	CH_4_	113.87	/	[138]
g-C_3_N_4_-RGO-NH_2_-MIL-125(Ti)	g-C_3_N_4_-RGO	CO	95.9	96.53	[139]
NMF/CsPbBr_3_	CsPbBr_3_	CO	50.7	/	[140]
TiO_2_@CoNi-MOF NTs	TiO_2_	CH_4_	41.65	93.2	[128]

## 6. MOF-Based Materials for CO_2_ Electrocatalytic Reduction

### 6.1. Pristine MOF

In electrocatalyst design, the primary objectives are to achieve exceptional adsorption capacity, ideal selectivity, high current density, rapid electron and mass transfer, and enduring stability. These attributes are essential for the development of efficient and robust electrocatalysts. One of the primary reasons for the high catalytic activity of MOFs is their uniformly dispersed active sites. The highly ordered structure in MOFs allows for the isolation and atomic dispersion of active sites, facilitating CO_2_ adsorption, transfer, and reduction. Moreover, by tuning the chemical structure of MOFs, various products such as CO, HCOOH, CH_4_, and C_2_H_4_ can be obtained. 

**Figure 21:**
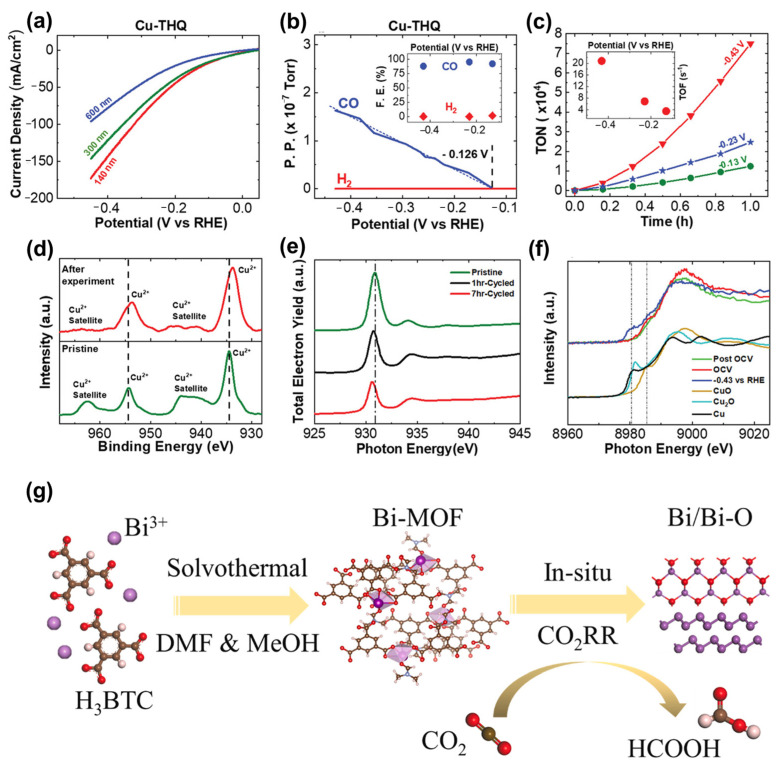
The performance of CO_2_ reduction at low overpotentials of Cu-THQ. (**a**) LSV results (scan rate of 50 mV s^−1^) for different sizes in 1 m choline chloride and 1 m KOH. (**b**) Differential electrochemical mass spectroscopy analysis for CO and H_2_ production during LSV experiment (scan rate of 1 mV s^−1^). The inset demonstrates the FE of CO and H_2_ at various potentials. (**c**) TONs for CO production during 1 h chronoamperometry experiments at three different potentials. The inset demonstrates TOFs at these potentials after 1 h. Structural changes analysis of Cu-THQ. (**d**) XPS results of Cu 2p region in Cu-THQ before and after 7 h reaction. (**e**) Cu-L_3_ XAS results of Cu-THQ before and after 7 h reaction displayed in total electron yield mode. (**f**) Operando XANES results of Cu-THQ at Cu K-edge. Reproduced from [141]. Copyright 2021, Wiley. (**g**) Schematic illustration of the synthetic process and reconstruction process of Bi-MOF during CO_2_RR. Reproduced from [144]. Copyright 2022, Elsevier.

CO and HCOOH are two readily accessible chemical products because only two electrons are required. Majidi et al. [141] fabricated 2D Copper tetrahydrixyquinone (Cu-THQ) nanoflakes (NFs) via a liquid-phase exfoliation method from bulk Cu-THQ (Cu_2_(C_6_O_6_)_2_). The average particle size of Cu-THQ NFs is approximately 140 nm, with an average statistical thickness of about 10.1 nm. LSV experiments were conducted using a Cu-THQ-coated gas diffusion electrode (GDE) as working electrode, Ag/AgNO_3_ as reference electrode, and platinum mesh as counter electrode, revealing that Cu-THQ NFs exhibit an ultra-high current density of 173 mA cm^−2^ at −0.45 V versus RHE toward CO selectivity, with an even lower onset potential of −0.126 V versus RHE and no detection of other carbon-based gas phase products (Figure 21a,b). Notably, Cu-THQ NFs also demonstrate considerable TON and TOF values of 7.49 × 10^4^ and 20.82 s^−1^ at −0.43 V versus RHE, respectively (Figure 21c), indicating their high catalytic activity. The possible change in Cu^2+^ chemical environment was studied by various techniques. X-ray Photoelectron Spectroscopy (XPS) and Cu L_3_-edge X-ray adsorption spectroscopy (XAS) results show that the peaks of the samples after reaction shifted to lower energy compared to the pristine sample (Figure 21d,e), signifying the changes in the chemical environment of Cu^2+^ species. To gain deeper insights into the alterations in electronic and chemical conditions, operando Cu K-edge X-ray absorption near edge spectroscopy (XANES) was conducted for Cu-THQ at various stages: open-circuit voltage (OCV), at −0.43 V versus RHE, and 12 min after the reaction ceased and returned to OCV. The XANES spectrum obtained at OCV reaffirmed the presence of Cu^2+^ state in the pristine compound. Upon applying a cathodic potential, the edge position shifts to a lower energy in comparison with the pristine state, suggesting the reduction of Cu^2+^ during electrocatalysis. After collecting data at −0.43 V versus RHE, the cell was reverted to open circuit to obtain XANES of the relaxed catalyst. The corresponding spectrum confirmed the formal Cu^2+^ state, with subtle deviations in line shape compared to the pristine compound (Figure 21f). The rapid return of the catalysts to Cu^2+^ suggests the generation of very small Cu clusters, but there were still subtle changes in the bonding of Cu and ligands.

Another two-electron product, HCOOH, was successfully obtained using an In-based MOF and Sn-based MOF [142,143]. These two materials both exhibit high electrocatalytic performance. It is worth noting that neither of the above two MOFs undergo structural changes during the reaction. However, some MOFs, such as Bi-based MOFs (Bi-MOFs) [144], are prone to reconstruction. The high selectivity of HCOOH is not facilitated by the Bi-MOF itself but rather by the reconstruction occurring at the Bi/Bi-O interface. During the electrocatalytic process, Bi-MOF is reduced to Bi and Bi_2_O_2.5_, and this hybrid Bi/Bi-O interface serves as the real active site for CO_2_RR (Figure 21g), which is beneficial for the adsorption of OCHO* intermediates, thus facilitating HCOOH formation.

Multi-electron product hydrocarbons hold significant value for industrial production. To achieve the synthesis of these deep reduction products, selecting appropriate metal nodes as the catalytic active sites is crucial. Among various metals, Cu stands out as the promising metal capable of producing deep reduction products. Different Cu valence states, including Cu(0), Cu(Ι), and Cu(II), exhibit varying binding energies toward reaction intermediates [145]. The structural reconstruction during the reaction plays a crucial role in hydrocarbon generation, although this may introduce instability in the catalysts, potentially impacting their performance over multiple cycles [146].

The performances of various pristine MOFs are illustrated in Table 6.

### 6.2. MOF Derivatives

Many researchers have utilized MOFs as templates to fabricate MOF derivatives. This approach holds promise, as it preserves the porous structure and atomically dispersed active sites of MOFs while concurrently enhancing their chemical and electrical properties. In the following sections, we will delve into the research progress on some key MOF derivatives. The performances of various MOF derivatives are illustrated in Table 7.

#### 6.2.1. MOF-Derived SACs

Ever since Qiao et al. [147] reported the successful assembly of isolated single Pt atoms anchored on an iron oxide support to achieve CO oxidation in 2011, the concept of SACs has gained increasing popularity in recent years. SACs are special class of catalysts wherein metal components are dispersed as individual atoms on a support material. In SACs, metal atoms exist in an isolated and dispersed form, different from the conventional NP catalysts. This unique structure imparts SACs with high catalytic activity and selectivity. Theoretically, the utilization of metal atoms can reach 100%. The tunable electronic structure and low coordinated configuration also make SACs possible to use in CO_2_RR [65]. 

**Figure 22:**
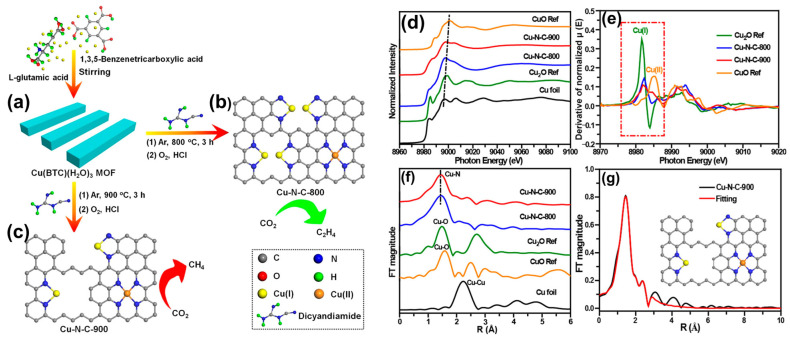
Synthetic procedure of Cu(BTC)(H_2_O)_3_ and Cu-N-C-800,900. (**a**) Cu(BTC)(H_2_O)_3_ as an MOF precursor. (**b**) Cu-N-C-800 and (**c**) Cu-N-900 derived from different pyrolysis temperatures. (**d**) Cu-K edge XANES spectra of the samples referring to Cu foil, Cu_2_O, and CuO. (**e**) First derivatives of the XANES region of the samples from (**d**). (**f**) FT-EXAFS Cu-K edge spectra of the samples referring to Cu foil, Cu_2_O, and CuO. (**g**) EXAFS *R*-space fitting curves of Cu–N–C-900 catalyst. Reproduced from [148]. Copyright 2020, American Chemical Society.

Owing to the atomically dispersed active sites separated by organic ligands, MOFs serve as ideal precursors for constructing SACs. MOF-derived SACs with different types of SA active centers have been extensively explored. Current research efforts focus on protecting the SA sites and optimizing their coordination environment. For example, MOF-derived SA Cu catalysts show great potential in the field of CO_2_ electroreduction to CO [148]. Two such catalysts, namely Cu-N-C-800 and Cu-N-C-900, were synthesized by pyrolyzing an N-doped MOF (Cu(BTC)(H_2_O)_3_) at 800 °C and 900 °C (Figure 22a), respectively. After pyrolysis, the organic ligands were transformed into an N-doped carbon framework with Cu, and excess Cu NPs were further removed by acid leaching. XAS is an indispensable technique for characterizing SACs. XANES and extended X-ray absorption fine structure (EXAFS) spectra confirm the oxidation state of Cu atoms and the absence of a Cu–Cu bond while affirming the presence of a Cu–N bond, indicating successful synthesis of SACs (Figure 22d–f). Different pyrolysis temperatures yield distinct structural configurations. In Cu-N-C-800, one Cu(Ι) atom coordinates with two pyridinic-N atoms, forming Cu-N_2_ sites, while one Cu(II) atom coordinates with four pyridinic-N atoms, resulting in Cu-N_4_ sites. Adjacent Cu-N_2_ sites are observed due to their proximity, whereas Cu-N_4_ sites remain isolated (Figure 22b). Conversely, in Cu-N-C-900, only isolated Cu-N_2_ and Cu-N_4_ sites are observed (Figure 22c), as confirmed by the fitting curves of EXAFS *R*-space (Figure 22g). The structural difference and N coordination numbers between Cu-N-C-800 and Cu-N-C-900 significantly impact product selectivity. Cu-N-C-800 exhibits superior FE_C2H4_ (24%) to FE_CH4_ (13.9%) at −1.4 V vs RHE, whereas Cu-N-C-900 exhibits a completely opposite trend, with CH_4_ being the dominant product. This distinction is strongly related to the coordination environment in Cu-N-C-800 and Cu-N-C-900, where the neighboring Cu-N_2_ sites favor C–C coupling for C_2_H_4_ production, while the isolated Cu-N_2_ and isolated Cu-N_4_ sites are more inclined to produce CH_4_. 

**Figure 23:**
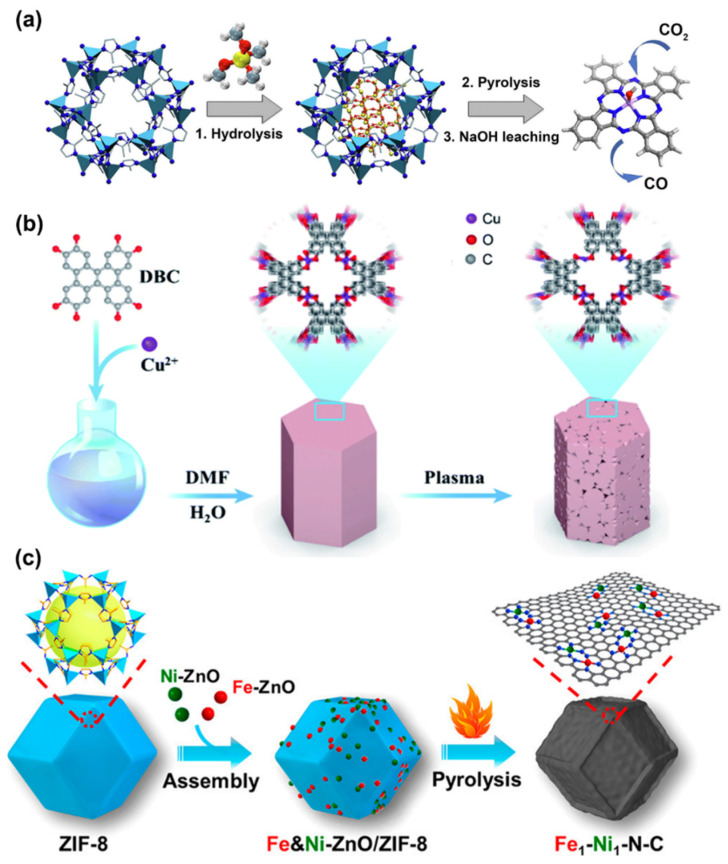
Synthetic procedure of (**a**) mesoNC-Fe catalyst by a SiO_2_-protective approach. Reproduced from [149]. Copyright 2019, Elsevier. (**b**) PA-CuDBC-1 catalyst by a plasma activated method. Reproduced from [150]. Royal Society of Chemistry. (**c**) Fe_1_-Ni_1_-N-C with Fe and Ni SA pairs. Reproduced from [151]. Copyright 2021, American Chemical Society.

The synthetic procedure for SACs is also a major concern worth discussing. Most SACs are obtained through high-temperature pyrolysis and acid etching processes, during which the aggregation of NPs may occur. Instead, a SiO_2_-protective approach is applied to prevent these issues [149]. Mesoporous nitrogen-doped carbon NPs with atomically dispersed iron sites (mesoNC-Fe) were achieved by using a three-step procedure (Figure 23a). Firstly, ZIF-8-Fe was suspended in tetramethyl orthosilicate (TMOS) solution, and further hydrolysis of TMOS resulted in the formation of ZIF-8-Fe@SiO_2_. Subsequently, ZIF-8-Fe@SiO_2_ was pyrolyzed at 900 °C under N_2_ atmosphere to obtain NC-Fe@SiO_2_. Lastly, NaOH solution was used for SiO_2_ template leaching. Hydrolysis of TMOS in the MOF framework before pyrolysis is beneficial for impeding aggregation of NPs, ensuring the generation of atomically dispersed iron active sites and maintaining high surface area and mesoporosity in the carbon matrix. Alternatively, Wei et al. [150] developed a novel approach for designing SACs without pyrolysis. They synthesized an SA Cu catalyst (PA-CuDBC-1, DBC: catecholate) with low-coordinated copper sites and a hierarchically porous structure via a plasma-activated strategy (Figure 23b). The plasma treatment has no detrimental effect on the performance of the catalyst. At −1.1 V versus RHE, the total FE of carbon-containing products could reach 96.5%, with a high partial current density of 47.8 mA cm^−2^. This work presents an avenue for substituting pyrolysis with a simple and effective approach under mild conditions. 

The potential synergistic effect of SA pairs, particularly for adjacent SA sites, should also be of great importance for catalysis. The adjacent Fe-N_4_ and Ni-N_4_ sites in Fe_1_-Ni_1_-N-C catalysts (Figure 23c) achieve an ultrahigh FE_CO_ of 96.2% at −0.5 V versus RHE, surpassing those of Fe_1_-N-C and Ni_1_-N-C catalysts [151]. This result suggests the synergy between SAs Fe and Ni in promoting CO_2_RR. DFT calculations reveal that the presence of SA Ni significantly reduces the free energy of COOH* formation on Fe sites and enhances the energy barrier for H_2_ production, promoting the CO_2_RR while simultaneously suppressing the Hydrogen Evolution Reaction (HER). In conclusion, the advancement of adjacent SA sites highlights their pivotal role in accelerating CO_2_RR. The catalytic efficacy of the individual SA sites is enhanced through the synergistic interaction between neighboring SA sites. In addition, SA alloy catalysts have demonstrated significant advantages in CO_2_ conversion towards HCOOH, suggesting that the development of MOFs in this direction opens up new possibilities for the electrochemical reduction of CO_2_ [152].

#### 6.2.2. MOF-Derived Metal Oxide

**Figure 24:**
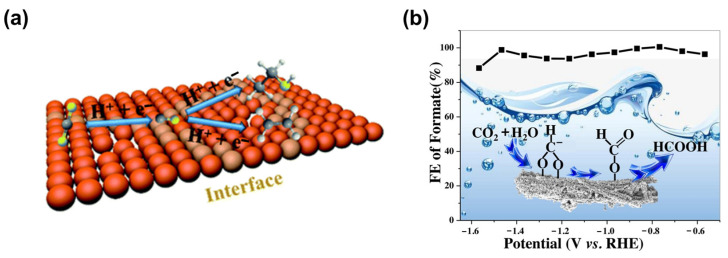
Schematic illustration of the CO_2_RR mechanism of (**a**) CuO nanowires. Reproduced from [153]. Copyright 2020, American Chemical Society. (**b**) The performance and mechanism of CuBi catalyst. Reproduced from [158]. Copyright 2021, Elsevier.

MOF-derived metal oxide exhibits enhanced intrinsic activity and conductivity in CO_2_RR. Yang et al. [153] employed copper-aspartic acid (Cu-ASP) nanofibers to prepare CuO nanowires through annealing Cu-ASP nanofibers in air. CuO nanowires achieve a C2 hydrocarbon FE of approximately 70% at −1.3 V versus RHE. Previous researchers have demonstrated that the (sub)surface of oxide-derived copper catalysts serves as a critical site for binding intermediates such as CO*, COH*, and CHO*, which are key intermediates for further transformation into C2 products [154,155,156]. Similarly, in this work, the extensive surface/interfaces of the oxide-derived metastable Cu during the reaction are advantageous for stabilizing the intermediates and facilitating the rapid production of C2 products (Figure 24a). 

Likewise, a novel Cu@Cu_2_O heterogeneous electrocatalyst was utilized to produce methanol by calcining Cu-BTC as a MOF precursor [157]. DFT results indicate that the Cu^0^/Cu^+^ interface, together with OH species on the surface, contribute to moderate CO* binding energy and strong H* adsorption, enhancing the hydrogenation of CO* to produce methanol. Therefore, the abundant surfaces/interfaces interaction between catalysts and adsorbates of the oxide-derived Cu catalysts give us insights into engineering MOF-derived metal oxide toward selective CO_2_RR. 

In addition to monometal oxides, Yang et al. [158] synthesized a CuBi bimetal catalyst derived from CuBi-MOF. The formation of Bi_2_Cu_2_O_4_ can enhance both the adsorption capacity of CO_2_ molecules and the charge transfer capability. For a wide potential window, CuBi exhibits a higher CO_2_ reductive activity toward HCOOH compared to MOF-derived monometallic catalysts and Bi_2_CuO_4_ without using MOF as a template (Figure 24b).

#### 6.2.3. MOF-Derived Metal/Carbon(Carbonate) Compound

**Figure 25:**
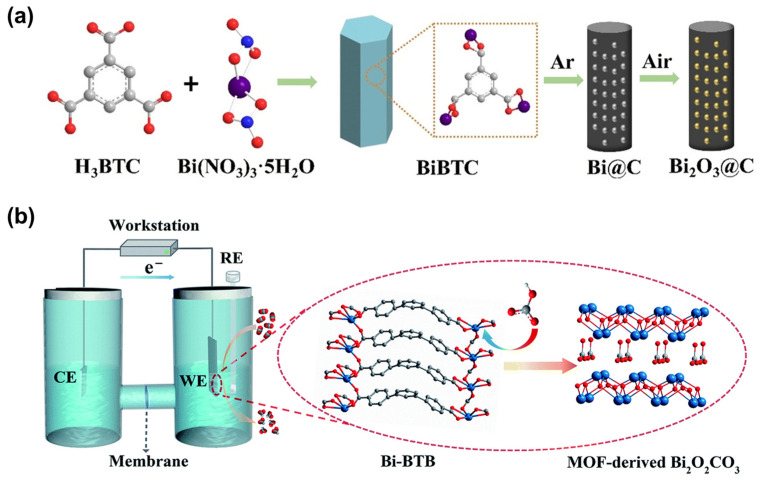
(**a**) Synthetic process of Bi_2_O_3_@C. Reproduced from [159]. Copyright 2020, Wiley. (**b**) Schematic illustration of the reconstruction process of Bi-BTB to form Bi_2_O_2_CO_3_ in HCO_3_^−^ solution. Reproduced from [160]. Copyright 2020, Royal Society of Chemistry.

Given the high conductivity of carbon, metal/carbon hybrids are also promising catalysts. In this example [159], Bi@C was first prepared by calcinating BiBTC at 800 °C in an Ar atmosphere. By further oxidizing Bi@C at 200 °C, the corresponding Bi_2_O_3_@C sample was obtained (Figure 25a). The electroreduction of CO_2_ was conducted both in H-type cell and flow cell configuration for comparison. In the H-type cell, the maximum FE_HCOOH_ reaches 92% at −0.9 V versus RHE, with a high HCOOH partial current density of 7.5 mA cm^−2^. In the flow cell configuration, Bi_2_O_3_@C performs better with a more positive onset potential, accompanied by FE_HCOOH_ of over 93% for a wide range of potentials and current density up to 200 mA cm^−2^ at −1.1 V versus RHE. Bi_2_O_3_@C also sustains its high FE_HCOOH_ and current density during long-term electrolysis, indicating excellent stability. The remarkable performance can be explained by the synergistic effect between carbon matrix and Bi_2_O_3_, where the carbon matrix helps to improve the activity and current density, and Bi_2_O_3_ promotes fast reaction kinetics and HCOOH selectivity. Moreover, subcarbonates can be derived from MOFs in HCO_3_^−^ solution. Yuan et al. [160] discovered that the Bi-O bonds in Bi–1,3,5-tris(4-carboxy-phenyl)benzene (Bi–BTB) were broken due to the effect of HCO_3_^−^, resulting in an in situ transformation into Bi_2_O_2_CO_3_ (Figure 25b). HCO_3_^−^ can break the Bi^3+^ and carboxylate bond in the original Bi-MOF and induce reconstruction to form Bi_2_O_2_CO_3_. 

#### 6.2.4. MOF-Derived N-Doped Porous Carbon (NPC)

**Figure 26:**
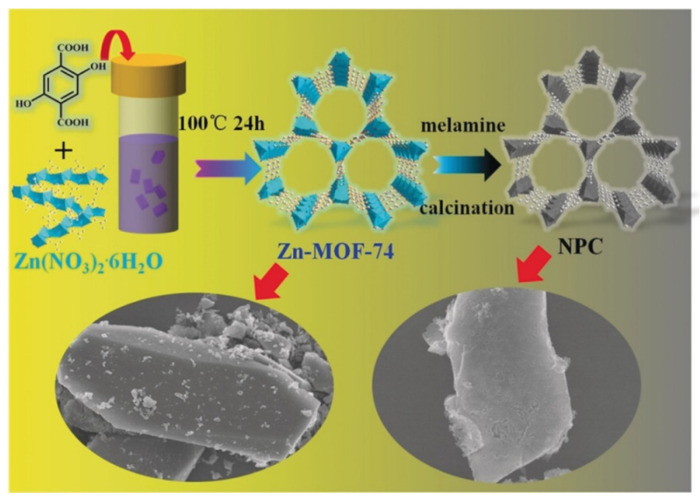
Synthetic procedure of NPC and the morphology of Zn-MOF-74 and NPC. Reproduced from [161]. Copyright 2019, Wiley.

Porous carbon has shown advantages in promoting CO_2_ adsorption and mass transfer. However, limited active sites have hindered its widespread use in catalysis. N species are important in CO_2_ reduction to CO, with distinct types including pyridinic nitrogen, pyrrolic nitrogen, graphitic nitrogen, and oxidized nitrogen. To this end, the development of NPC has attracted great interest as a promising candidate. Ye et al. [161] reported an NPC derived from an oxygen-rich MOF precursor, Zn-MOF-74, with melamine as N source (Figure 26). By adjusting calcination conditions, the types and contents of N, along with pore size distribution, can be controlled. By increasing the temperature from 900 °C to 1000 °C, the abundance of active N species increases, alongside a higher proportion of opened pore space. As a result, the optimized NPC achieves a high FE_CO_ of 98.4% at −0.55 V versus RHE, with a small onset potential of −0.35 V. The exceptional performance is attributed to the presence of active N sites and large pore size. These features lower the energy barriers of the potential limiting steps for CO_2_RR and make CO_2_ more accessible to the active sites. Additionally, the author also prepared a porous carbon without N doping, and the result shows that both FE_CO_ and current density are inferior to that of NPC, further confirming the positive role of N species.

**Table 6:** Pristine MOFs for CO_2_ electrocatalytic reduction and their performance.

MOF	Main Product	Faradic Efficiency (%)	Potential (V vs. RHE)	Ref.
Cu_2_(CuTCPP)	HCOOH	68.4	−1.55 (V vs. Ag/Ag^+^)	[162]
In-BDC	HCOOH	88	−0.669	[142]
Bi-MOF	HCOOH	92	−0.64	[144]
Sn-MOF	HCOOH	92	−1.2	[143]
Sn-MOFPb_5_	HCOOH	94.6	−1.1	[143]
Cu-THQ	CO	91	−0.45	[141]

**Table 7:** MOF derivatives for CO_2_ electrocatalytic reduction and their performance.

Material	MOF Precursor	Main Product	Faradic Efficiency (%)	Potential (V vs. RHE)	Ref.
MOF-derived SACs					
mesoNC-Fe	ZIF-8-Fe	CO	85	−0.73	[149]
Ni_SA_-N_2_-C	MgNi-MOF-74	CO	98	−0.8	[163]
Fe_1_−Ni_1_−N−C	Fe&Ni-ZnO/ZIF-8	CO	96.2	−0.5	[151]
PA-CuDBC-1	CuDBC	CH_4_	75.3	−1.1	[150]
Ni-N-C	ZIF-8	CO	99	−0.68	[164]
Ni1-N-C (Cl)	ZIF-8	CO	94.7	−0.7	[165]
Cu−N−C-800	Cu(BTC)(H_2_O)_3_	C_2_H_4_	24.8	−1.4	[148]
Cu−N−C-900	Cu(BTC)(H_2_O)_3_	CH_4_	38.6	−1.4	[148]
2Bn-Cu@UiO-67	UiO-67	CH_4_	81	−1.5	[166]
MOF-derived metal compound					
CuBi75	CuBi-MOF	HCOOH	100	−0.77	[158]
Bi_2_O_3_@C-800	BiBTC	HCOOH	92	−0.9	[159]
OD-Cu-3	Cu-ASP	C_2_H_4_	45	−1.3	[153]
Cu@Cu_2_O-400	Cu-BTC	CH_3_OH	45	−0.7	[157]
BiIn_5_-500@C	Bi-MOF	HCOOH	97.5	−0.86	[167]
Bi_2_O_2_CO_3_	Bi-BTB	HCOOH	96.1	−0.669	[160]
MOF-derived NPC					
NPC-1000	Zn-MOF-74	CO	98.4	−0.55	[161]

## 7. MOF-Based Materials for CO_2_ Hydrogenation

### 7.1. RWGS Reaction

CO serves as a pivotal precursor in chemical synthesis, particularly in Fischer–Tropsch Synthesis. This process is instrumental in the transformation of CO into a spectrum of liquid hydrocarbon fuels and an array of chemical products. An aerosol-assisted method was used to synthesize a Cu-based MOF (HKUST-1) supported on CeO_2_ NP clusters. This composite was then utilized to fabricate MOF-derived Cu@CeO_2_ hybrid nanomaterials for the RWGS reaction [168]. The study reveals that by reducing the Cu/Ce atomic ratio in the hybrid material, both the metal surface area and dispersion are significantly enhanced. The incorporation of CeO_2_ NP clusters is shown to improve the activity and stability of the MOF-derived nanostructured catalysts. The catalysts exhibit high selectivity and operational stability for RWGS, achieving superior catalytic activity at relatively low temperatures, exemplified by a TOF of 0.1635 s^−1^ at 400 °C. Gaje et al. [169] reported the development of a novel composite catalyst, derived from the polyoxometalate-HKUST-1 complex, forming a nanostructured Na-Cu-Mo_2_C material. Utilizing a unique in situ carburization method of MOF, they immobilized Cu metal NPs uniformly dispersed onto Mo_2_C. This newly developed catalyst exhibits superior performance in the RWGS reaction, with a CO production rate of 3230 mmol g_cat_^−1^ h^−1^ and 100% CO selectivity. Notably, the catalyst retains over 80% of its initial activity even after a 250 h stability test.

### 7.2. CO_2_ Methanation

CO_2_ methanation is a key technology for integrating renewable energy with the existing natural gas infrastructure and for creating a sustainable energy system. Chen et al. [170] reported the development of a Zr-based MOF (MOF-545: Zr_6_O_4_(OH)_4_(TCPP)_x_) composite for CO_2_ methanation. They initially investigated the performance of MOF-545 and Cu-modified MOF-545 (MOF-545(Cu)) as support catalysts under catalytic conditions, and found that the MOF could be transformed into a catalytically active material under high-temperature conditions.

Subsequently, Ni@MOF-545 catalysts were prepared using impregnation (IM) and double solvent (DS) methods, followed by dry reduction under hydrogen to immobilize Ni NPs. The results show that the Ni@MOF-545 catalyst, prepared using the DS method, exhibits the highest catalytic activity, with a methane production rate of 595 mmol_CH4_ g_Ni_^−1^ h^−1^ and a CO_2_ conversion of 60% after approximately 3 h, along with 100% CH_4_ selectivity.

### 7.3. CO_2_ Methanolation

Given the extensive range of applications of CH_3_OH in comparison to CO and CH_4_, there is a significant market demand for this compound. Consequently, CO_2_ hydrogenation into methanol is regarded as a process with considerable economic potential and profitability. However, despite the aforementioned advantages of CH_3_OH production, it is essential to consider the technical challenges associated with the synthesis process, including the selection of catalysts, optimization of reaction conditions, and energy efficiency. Moreover, the toxicity and corrosiveness of CH_3_OH must be carefully addressed in its applications.

**Figure 27:**
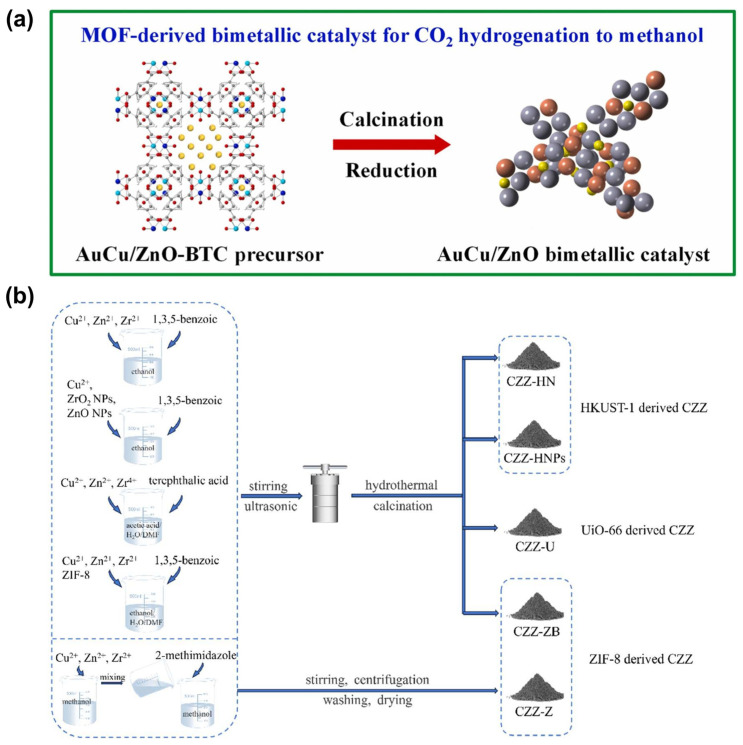
Schematic illustration of the synthetic procedure of (**a**) AuCu/ZnO catalysts from a MOF precursor. Reproduced from [171]. Copyright Elsevier, 2024. (**b**) Five types of CZZ catalysts from different MOF precursors. Reproduced from [172]. Copyright Elsevier, 2024.

The Cu/ZnO/Al_2_O_3_ catalyst has been commercially used in the transformation of CO and H_2_ into CH_3_OH. The tri-component catalyst, with Cu as the active phase, ZnO as the promoter, and Al_2_O_3_ as the support, exemplifies a synergistic ensemble that endows the catalyst with remarkable activity and selectivity. The design of MOF-based materials for CO_2_ methanolation is progressing toward this end. Three types of novel AuCu/ZnO bimetallic catalyst, namely AuCu/ZnO-BTC, AuCu/ZnO-BDC, and AuCu/ZnO-MOF-74, were prepared through a straightforward hydrothermal method from various MOF precursors [171] (Figure 27a). Under conditions of 250 °C and 3 MPa, the AuCu/ZnO-BTC catalyst demonstrates the highest activity, with a space–time yield (*STY*_MeOH_) of 359 g_MeOH_ kg_cat_^−1^ h^−1^, indicating high activity in the CO_2_ hydrogenation reaction for CH_3_OH production. Compared to the other two catalysts, AuCu/ZnO-BTC has a higher specific surface area, smaller metal particle size, more oxygen vacancies, stronger metal–support interactions, and a larger number of medium basic sites. As confirmed by in situ diffuse reflectance infrared Fourier transform spectroscopy (DRIFTS) studies, these features allow easy generation of *HCOO and bridged-methoxy (*b-OCH_3_) species, which may work together synergistically to enhance CH_3_OH synthesis activity. Wu et al. [172] utilized different MOF precursors (HKUST-1, ZIF-8, and UiO-66) to synthesize five types of MOF-derivative Cu-ZnO-ZrO_2_ (CZZ) catalysts through a top–down approach (Figure 27b). These were compared with a conventionally prepared catalyst (CZZ-CP) by co-precipitation method. In the CO_2_ hydrogenation reaction, MOF-derived catalysts show varying CO_2_ conversion rates and methanol selectivities compared to CZZ-CP. Notably, the catalyst derived from HKUST-1 demonstrates superior CH_3_OH synthesis activity and selectivity due to its enhanced metal–oxide interactions and higher number of surface Cu sites. This indicates that CO_2_ hydrogenation activity is related to the number of surface Cu sites, and different Cu sites influence the product distribution. The catalyst derived from HKUST-1 shows higher *STY*_MeOH_ and TOF due to its higher Cu^+^ content and reduction properties. 

Despite constructing MOF derivatives for CO_2_ methanolation, the use of MOF as a support also shows promise. Vali et al. [173] presented a study on the synthesis and application of Cu/ZnO/CeO_2_, supported on the MOF-5 (also denoted as HKUST-1). The Cu/ZnO/CeO_2_ nanocomposite supported on MOF-5 enhances the dispersion of active sites and controls NP agglomeration during synthesis. This approach addresses the issue of low surface area in commercial Cu/ZnO/Al_2_O_3_ catalysts caused by NP agglomeration. The Cu/ZnO/CeO_2_@MOF-5 catalyst demonstrates higher CH_3_OH selectivity compared to commercial catalysts, attributed to the interfacial sites generated between MOF-5 and Cu/ZnO, favoring CH_3_OH synthesis over CO. CeO_2_ as a support for Cu/ZnO instead of Al_2_O_3_ results in smaller particle size and superior dispersion of Cu active sites. The oxygen vacancies generated on the Cu/CeO_2_ interface due to metal–support interactions are found to enhance Cu dispersion and stabilize key reaction intermediates, leading to higher selectivity. This article illustrates an effective modification of traditional catalysts and elucidates the advantages of utilizing MOFs as a support.

## 8. MOF-Based Materials for Dual Functionalities

An innovative approach to CCU, known as Reactive Capture (RC), integrates the processes of CO_2_ capture and conversion. This approach is designed to reduce the energy and capital costs in the production of valuable products from dilute CO_2_ streams such as air or flue gas [174]. The direct conversion of captured CO_2_ into value-added products eliminates the need for dedicated CO_2_ desorption and compression steps, thereby reducing energy intensity and capital expenditure. The design of MOF-based materials for both CO_2_ capture and conversion to form bifunctional materials shows a great application prospect. The bifunctional materials are important in reducing the operational costs and ensuring the longevity of the materials in industrial applications. These advantages make the bifunctional materials highly suitable for large-scale implementation in various industrial sectors, particularly those with high CO_2_ emissions, offering a sustainable solution for carbon management and chemical production.

**Figure 28:**
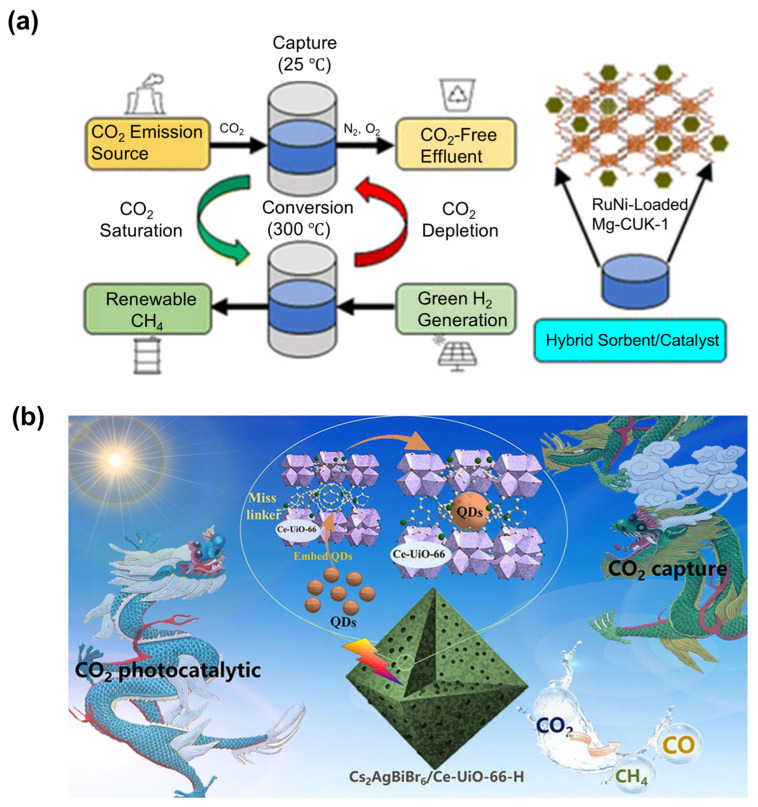
The bifunctional materials of (**a**) RuNi-loaded Mg-CUK-1 for both CO_2_ capture and conversion into CH_4_. Reproduced from [175]. Copyright 2022, Royal Society of Chemistry. (**b**) Cs_2_AgBiBr_6_/Ce-UiO-66-H for both CO_2_ capture and conversion into valuable chemicals. Reproduced from [176]. Copyright 2022, Elsevier.

Timothy et al. [175] present an innovative method for the development of a hybrid sorbent/catalyst material for the efficient CO_2_ capture and conversion into methane (Figure 28a). The material, named 0.3Ru2.7Ni Mg-CUK-1, integrates an MOF, Mg-CUK-1 (Mg_3_(*μ*_3_OH)_2_(2,4−pdc)_2_), with bimetallic RuNi NPs. This hybrid material leverages the advantages of Mg-CUK-1 for CO_2_ adsorption and the catalytic properties of Ru and Ni for the hydrogenation of CO_2_. The low loading of nanocatalysts on the MOF preserves substantial CO_2_ uptake capacity. The excellent CO_2_ conversion ability is further proven by the hydrogenation reaction due to the presence of Ru and Ni. A significant aspect of the study is the assessment of catalyst performance under oxygen exposure, which is critical for practical applications. The study found that Ru-loaded Mg-CUK-1 exhibited oxygen tolerance, maintaining CH_4_ generation over multiple cycles. The introduction of Ru helps to maintain the catalytic activity of Ni in the presence of oxygen. This research highlights that the MOF hybrid can be used as an effective bifunctional material for both CO_2_ capture and conversion.

In addition, an innovative class of bifunctional materials has been developed which incorporates Cs_2_AgBiBr_6_ QDs into the framework of Ce-UiO-66-H. This composite significantly enhances the CO_2_ adsorption capacity and effectively harnesses solar energy to drive the transformation of CO_2_ into useful chemical fuels (Figure 28b) [176]. The integration of Cs_2_AgBiBr_6_ QDs within the Ce-UiO-66-H matrix not only boosts the CO_2_ uptake owing to the larger surface area and the presence of more active sites but also facilitates the separation of photogenerated charge carriers, highlighting the synergistic effect of the composite structure. This article presents a significant step forward in the creation of a novel material for CO_2_ capture and photocatalytic conversion, showcasing the potential of combining inorganic SCs with MOFs for enhanced CO_2_ capture and solar-energy-driven CO_2_ reduction to valuable chemicals.

## 9. Conclusions 

Recent decades have witnessed the rapid development of MOF-based materials. The continuous advancement of MOFs offers boundless opportunities for CO_2_ capture and conversion, crucial for mitigating greenhouse gas emissions and addressing climate change. The inherent advantages of MOFs make them highly promising materials. The large specific surface area and porous structure facilitate efficient CO_2_ adsorption. In addition, their tunable chemical structure allows for various modification strategies aimed at enhancing MOF properties. These include the formation of open metal sites and the introduction of functional groups to enhance affinity with CO_2_, thus promoting interactions between CO_2_ molecules and the framework. The precise architectural arrangement within MOFs ensures a uniform distribution of catalytically active sites, thereby granting them exceptional catalytic efficacy for CO_2_ reduction. Additionally, MOFs serve as ideal templates and precursors for fabricating MOF composites or derivatives. The resultant materials not only inherit the corresponding merits of MOFs but also generate new benefits. Research aimed at improving CO_2_ capture and conversion capacity remains ongoing, with the selection of appropriate MOFs being crucial for achieving desired results.

In this article, we have first introduced the processes and mechanisms of CO_2_ capture and conversion. We then provided an overview of recent progress in MOF-based materials for CO_2_ capture and conversion, including CO_2_ adsorption and separation, photocatalytic reduction, electrocatalytic reduction, CO_2_ hydrogenation, and dual functionalities. We highlighted that the physical structures and chemical compositions of MOFs are key factors for CO_2_ adsorption and separation. The performance and selectivity of CO_2_ catalytic reduction are strongly associated with the electron transfer pathways and involvement of intermediates. All the illustrated examples provided us with deep insights into synthesizing highly active and stable MOF-based materials. 

## 10. Future Directions toward Industrial Applications 

The next frontier in the advancement of MOFs for industrial applications lies in targeted enhancements that address specific industry needs. Here, we propose a focused strategy for the development of MOF-based materials for CO_2_ capture and conversion.

1. Targeted synthesis for specific industries: The development of MOFs tailored for CO_2_ capture and separation in the petrochemical industry, for instance, requires materials that can selectively adsorb and desorb CO_2_ under process conditions. Future research should concentrate on the synthesis of MOFs with pore sizes and structure chemistries optimized for the capture and separation of CO_2_ from CH_4_, N_2_, and C_2_H_2_. In addition, a comprehensive study on the dynamic adsorption behavior of MOFs should be undertaken.
2. Enhancing MOF stability under industrial conditions: For MOFs to be viable in industrial settings, they must exhibit enhanced stability in the presence of moisture, heat, and mechanical stress. Future directions should include the design of MOFs with cross-linked structures or the incorporation of inorganic elements to bolster their robustness.
3. Catalytic MOFs for chemical production: The integration of catalytic sites within MOFs opens up avenues for one-pot synthesis and cascade reactions. The future will see the design of MOFs that can serve as heterogeneous catalysts for CO_2_-related chemical production, with a focus on optimizing catalytic activity, selectivity, and recyclability. A design strategy oriented toward MOFs with multiple catalytic sites that can perform multistep reactions within a single framework is highly desirable.
4. Dual functionalities for both CO_2_ capture and conversion: Dual-functional MOFs offer a one-step solution that combines adsorption and catalytic conversion within the same material. This not only reduces the need for separate processes and equipment but also potentially lowers the overall cost and complexity of CO_2_ management. By capturing CO_2_ and converting it into valuable chemicals or fuels in a single operation, dual-functional MOFs can increase the utilization rate of CO_2_, contributing to a circular carbon economy.
5. Regulatory compliance and safety: As MOFs move toward industrial applications, compliance with safety regulations is imperative. Future work must include comprehensive safety assessments, including toxicity and environmental impact, to ensure that MOFs meet industry standards.
6. Lifecycle and cost analysis: A thorough analysis of the lifecycle costs associated with MOF production, use, and disposal is essential. Future directions should include the development of cost models and strategies for recycling and reusing MOF materials to minimize waste and environmental impact.
7. Industrial pilot studies: Transitioning from lab scale to industrial scale requires pilot studies to evaluate the performance of MOFs in real-world conditions. Future efforts should focus on scaling up MOF production and conducting pilot trials in collaboration with industry partners.

In summary, the future of MOF-based materials in CO_2_ capture and conversion will hinge on the optimization of selectivity, kinetics, activity, stability, and economical efficiency, with close collaboration between material scientists, chemical engineers, and industry stakeholders to translate these advanced materials into practical solutions.

## Figures and Tables

**Table 1:** Various products of CO_2_ reduction and the corresponding reduction potentials.

Reactions	E^0^_redox_ (vs.NHE)/V
CO_2_ + 2H^+^ + 2e^−^ → HCOOH	−0.61
CO_2_ + 2H^+^ + 2e^−^ → CO + H_2_O	−0.53
CO_2_ + 4H^+^ + 4e^−^ → HCHO + H_2_O	−0.48
CO_2_ + 6H^+^ + 6e^−^ → CH_3_OH + H_2_O	−0.38
CO_2_ + 8H^+^ + 8e^−^ → CH_4_ + 2H_2_O	−0.24
2CO_2_ + 12H^+^ + 12e^−^ → C_2_H_4_ + 4H_2_O	−0.34
2CO_2_ + 12H^+^ + 12e^−^ → C_2_H_5_OH + 3H_2_O	−0.33
2CO_2_ + 14H^+^ + 14e^−^ → C_2_H_6_ + 4H_2_O	−0.27

## Nomenclature

BTC	1,3,5-benzene-tricarboxylic acid	bpy	4,4′-bipyridine
BPDC	5,5′-dicarboxylate-3,3′-dipyridine	TPDC	terphenyl-4,4″-dicarboxylate
ZIF	zeolitic imidazolate framework	MeIm	methylimidazole
ZIF-8	Zn(MeIm)_2_	Hmim	2-methylimidazole
ZIF-67	Co(Hmim)_2_	DMF	N,N-Dimethylformamide
MIL	Materials of Institut Lavoisier	BMI	1-butyl-3-methylimidazolium
MIL-101	Cr_3_F(H_2_O)_2_O(BDC)_3_·nH_2_O	BDC	1,4-benzenedicarboxylate
MIL-125	Ti_8_O_8_(OH)_4_–(O_2_C–C_6_H_4_–CO_2_)_6_	UiO	University of Oslo
UiO-66	Zr_6_O_6_(OH)_4_(BDC)	UiO-67	Zr_6_O_6_(OH)_4_(BPDC)
UiO-68	Zr_6_O_6_(OH)_4_(TPDC)	TCA^3–^	4,4′,4″-tricarboxyltriphenylamine
MOF-808	[Zr_6_O_4_(μ_3_-OH)_4_(OH)_6_(H_2_O)_6_(BTC)_2_]·nH_2_O	HKUST	Cu_3_(BTC)_2_
MBA	2-(5′-methyl-[2,2′-bipyridine]-5-yl)acetic acid	OD	oxide-derived
ABPY	4,4′-(9,10-anthracenediyl)bis-Pyridine	ASP	aspartic acid
H_4_OATA	N,N′-bis(3,5-dicarboxyphenyl)oxalamide	PA	plasma-activated
F-H_2_tzba	2-F-4-(1H-tetrazol-5-yl) benzoic acid	NPC	N-doped porous carbon
(H_2_PhTz)Br	5-carboxy-3-(4-carboxybenzyl)thiazolium bromide	DBC	catecholate
H_2_bpydc	2,2′-bipyridine-5,5′-dicarboxylic acid	THQ	tetrahydroxyquinone
dobpdc^4–^	4,4′-dioxidobiphenyl-3,3′-dicarboxylate	dps	4.4′-dipyridylsulfide
NNU-55-M	[M_3_(5-AD)_4_(CO_2_)_2_] (CH_3_COO^−^)_2_ (M: Co, Ni, 5-AD: 5-Azabenzimidazole)		
NNU-31-M	[Fe_2_M(μ_3_-O)(TCA)_2_(H_2_O)_3_] (M: Co, Ni, Zn)	PT	phenanthroline dibenzoate
PCN	porous coordination network	ABTC:	3,3′,5,5′-azobenzenetetracarboxylate
PCN-250	consisting of Fe_3_-μ_3_-oxo clusters and tetratopic azobenzene-based ABTC ligand	BTB	1,3,5-tris(4-carboxy-phenyl)benzene
aip	5-aminoisophthalic	Tripp	2,4,6-tris(4-pyridyl)pyridine
dppe	1,2-bis(diphenylphosphino)ethane		
NHC	N-heterocyclic carbenes	MUF	Massey University Framework
NMF	[Ni(phen)(oba)]_n_·0.5_n_H_2_O (phen: 1,10-phenanthroline, oba: 4,4′-oxybis (benzoate))	MOF-177	Zn_4_O(BTB)_2_
pdc	pyridine dicarboxylate		
MOF-11	Cu_2_(ATC)·6H_2_O	H_2_L	2-(imidazol-1-yl)terephthalic acid
BTC	1,3,5-benzenetricarboxylate	DOBDC	2,5-dioxido-1,4-benzenedicarboxylate
MOF-74	consisted of transition-metal cations (M) (M: Zn^2+^, Mg^2+^, Co^2+^, Cd^2+^, Mn^2+^, Fe^2+^, or Ni^2+^) and the dobdc^4−^ or dhtp^4−^ (2,5-dihydroxyterephthalate) ligand		
TAPP	4,4′,4″-(1,3,5-triazine-2,4,6-triyl) trianiline	DPA	2,5-dibromo-1,4-diformylbenzen
H_6_TCPP	meso-tetra(carboxyphenyl)porphyrin	ATC	1,3,5,7-Adamantane Tetracarboxylate
DHBDC	2,5-dihydroxybenzene-1,4-dicarboxylic acid	CO_2_RR	electrochemical CO_2_ reduction
